# Lactate-induced MBD2 K186 lactylation promotes YAP1-dependent myofibroblast-like remodeling of Müller cells in diabetic retinopathy

**DOI:** 10.1186/s43556-026-00605-2

**Published:** 2026-10-06

**Authors:** Xindan Xing, Yan Jiang, Junwei Fang, Xin Shi, Qian Zhu, Yinchen shen, Li Su, Xinyi Liu, Hanying Wang, Jin Wei, Yuan Qu, Kangjia Lv, Yu Xiao, Jingtong Xie, Xiaoxin Liu, Hancong Wan, Tian Niu, Kun Liu

**Affiliations:** Department of Ophtalmology, Shanghai General Hospital, National Clinical Research Center for Eye Diseases, Shanghai Clinical Research Center for Eye Diseases, Shanghai Key Clinical Specialty, Shanghai Key Laboratory of Ocular Fundus Diseases, Shanghai Engineering Center for Visual Science and Photomedicine, Shanghai Engineering Center for Precise Diagnosis and Treatment of Eye Disease, Shanghai, China

**Keywords:** Proliferative diabetic retinopathy, Müller cells, Myofibroblast-like phenotype, Lactate, Protein lactylation, MBD2–YAP1 signaling

## Abstract

Tractional retinal detachment caused by fibroproliferative membrane formation is a vision-threatening complication of proliferative diabetic retinopathy (PDR). Müller cells can acquire myofibroblast-like features during retinal fibroproliferative remodeling, yet the metabolic mechanisms governing these phenotypic changes remain poorly understood. Here, we report that lactate is a metabolic regulator of myofibroblast-like remodeling in Müller cells. Metabolomic profiling revealed elevated vitreous lactate levels in patients with PDR. Consistently, lactate concentrations were increased in the vitreous and retinal tissues of diabetic rats, whereas high-glucose exposure promoted lactate accumulation in primary Müller cells. Increased lactate availability enhanced Müller cell proliferative activity and migration, as well as the expression of myofibroblast-associated proteins, including α-smooth muscle actin, smooth muscle protein 22, and collagen I, whereas oxamate, an inhibitor of lactate dehydrogenase, attenuated these responses. Lactylome profiling and molecular validation revealed K186 of methyl-CpG-binding domain protein 2 (MBD2) as a functionally relevant lactylation site. RNA sequencing of high-glucose-treated Müller cells expressing MBD2-K186R versus MBD2-WT revealed reduced YAP1 expression in the K186R group, suggesting that YAP1 is a downstream effector of MBD2 K186 lactylation. Mechanistically, increased lactate availability enhanced MBD2 K186 lactylation and YAP1 activation, as evidenced by reduced inhibitory phosphorylation of YAP1 and increased nuclear accumulation of YAP1. MBD2 K186R attenuated YAP1 activation and myofibroblast-like changes, whereas YAP1 overexpression partially reversed these effects. Collectively, these findings reveal a lactate–MBD2 K186 lactylation–YAP1 signaling axis that promotes myofibroblast-like remodeling of Müller cells and may contribute to fibroproliferative pathology in PDR.

**Supplementary Information:**

The online version contains supplementary material available at https://doi.org/10.1186/s43556-026-00605-2.

## Introduction

Diabetic retinopathy (DR) is among the most common microvascular complications of diabetes and remains a major cause of vision impairment worldwide. Proliferative diabetic retinopathy (PDR), the advanced and vision-threatening stage of DR, is characterized by pathological neovascularization accompanied by fibrovascular membrane formation [1–4]. Although anti-vascular endothelial growth factor (VEGF) therapy has substantially improved the control of pathological retinal neovascularization, fibroproliferative remodeling remains difficult to manage and can lead to persistent vitreoretinal traction, tractional retinal detachment, and irreversible visual loss [4, 5]. Thus, identifying the cellular and molecular mechanisms that drive fibrovascular remodeling is important for developing complementary therapeutic strategies that extend beyond vascular-targeted intervention.

Müller cells, the principal macroglia of the retina, are essential for maintaining neuronal metabolism, neurotransmitter recycling, ion and water balance, and retinal microenvironmental homeostasis [6, 7]. Under diabetic stress, Müller cells undergo reactive gliosis, inflammatory activation, metabolic disturbance, and extracellular matrix remodeling. Furthermore, accumulating evidence suggests that they can acquire contractile and extracellular matrix-producing properties that may contribute to fibrovascular membrane formation [6–11]. Such myofibroblast-like remodeling is a multidimensional process rather than a phenotype defined by a single marker. Increased α-smooth muscle actin (α-SMA) and smooth muscle protein 22α (SM22α) reflect the acquisition of contractile characteristics, whereas increased COL1A1 reflects enhanced extracellular matrix production [10–12]. These changes support the acquisition of myofibroblast-like features without necessarily implying complete lineage conversion. Although TGF-β signaling is a well-established inducer of these phenotypic alterations in Müller cells [10, 11], the metabolic signals that initiate or sustain this remodeling under diabetic conditions remain incompletely understood.

Metabolic reprogramming has increasingly been recognized as an important determinant of cellular plasticity and fibrotic disease progression. Lactate, traditionally viewed as a terminal product of glycolysis, is now recognized as a bioactive metabolite capable of regulating cellular behavior through receptor-mediated signaling and intracellular posttranslational modifications [13–15]. Müller cells exhibit a highly glycolytic metabolic profile and participate substantially in retinal lactate production and utilization [13]. In addition, the lactate-responsive receptor hydroxycarboxylic acid receptor 1 (HCAR1/GPR81) is expressed in Müller cells and participates in retinal vascular regulation [16]. In several nonocular fibrotic disorders, increased glycolytic flux and lactate accumulation have been linked to myofibroblast activation and extracellular matrix production [17, 18]. However, whether elevated lactate directly promotes myofibroblast-like remodeling of Müller cells and thereby contributes to fibroproliferative pathology in PDR remains unclear.

Lysine lactylation provides a potential mechanism through which altered lactate availability may be translated into sustained cellular reprogramming. Initially identified as a histone modification that links glycolytic metabolism to transcriptional regulation, lactylation is known to occur on nonhistone proteins and can influence protein stability, interactions, localization, and function [19–22]. Nevertheless, the functional targets and biological consequences of protein lactylation in retinal Müller cells remain largely unexplored. Methyl-CpG-binding domain protein 2 (MBD2) is a chromatin-associated methyl-CpG-binding protein involved in transcriptional regulation and has been implicated in diabetic retinal injury as well as inflammatory and fibrotic responses in nonocular systems [23–26]. Yes-associated protein 1 (YAP1), a major effector of the Hippo pathway, regulates proliferation, migration, mechanotransduction, and fibrotic gene programs [27–31], and YAP/TEAD signaling has been implicated in reactive Müller cell responses and experimental retinal fibrosis [28, 29]. These observations raise the possibility that lactate-dependent protein modification may intersect with MBD2- and YAP1-associated regulatory mechanisms during Müller cell remodeling, although the molecular connection among these processes has not been established.

Accordingly, the objective of this study was to determine whether elevated lactate promotes myofibroblast-like remodeling of retinal Müller cells and to identify the lactylation-dependent molecular mechanisms underlying this response. We hypothesized that increased lactate availability in the diabetic retinal microenvironment promotes Müller cell remodeling through protein lactylation and downstream profibrotic signaling. To test this hypothesis, we combined analyses of vitreous samples from patients with PDR and diabetic retinal models with primary Müller cell experiments, lactylome profiling, site-directed mutagenesis, transcriptomic analysis, protein interaction assays, and functional rescue experiments. We found that the level of lactate was elevated under PDR-associated conditions and promoted the proliferation and migration of Müller cells and the expression of contractile and extracellular matrix-associated markers. Unbiased lactylome profiling revealed MBD2 as a nonhistone lactylated protein and suggested that K186 is a functionally important site, whereas subsequent transcriptomic and functional analyses revealed that YAP1 is a downstream mediator of K186-dependent MBD2 regulation. K186R mutation markedly reduced MBD2 lactylation and attenuated YAP1 activation and myofibroblast-like remodeling, and these effects were partially reversed by YAP1 overexpression. Together, these findings reveal a previously unrecognized lactate-responsive MBD2–YAP1 regulatory mechanism and provide a potential molecular link between diabetes-associated metabolic reprogramming and fibroproliferative remodeling in PDR.

## Results

### Intraocular lactate accumulation is associated with myofibroblast-like changes in PDR

**Fig. 1:**
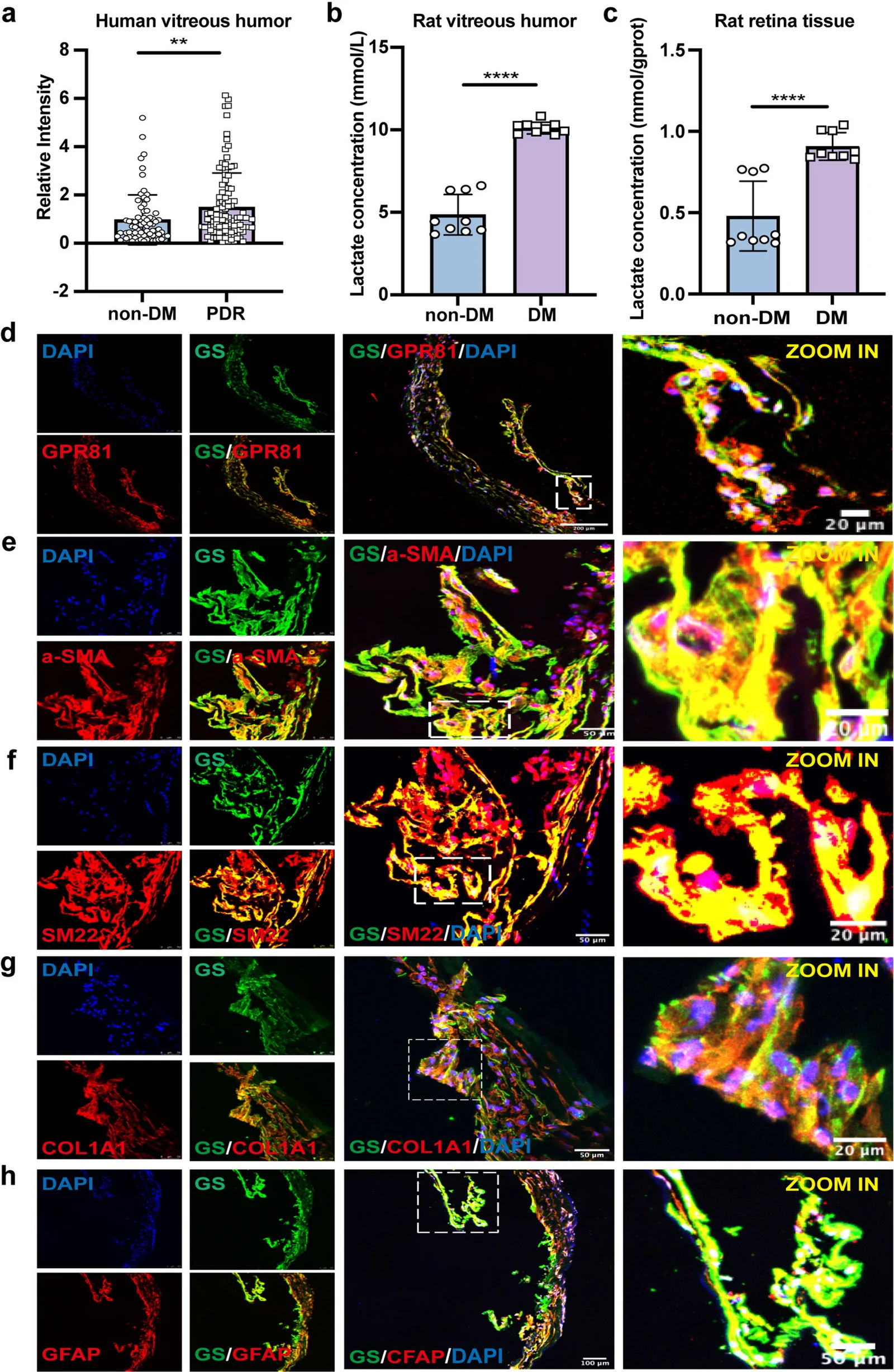
Intraocular lactate accumulation is associated with myofibroblast-like features in proliferative diabetic retinopathy.** a** Relative lactate abundance in human vitreous samples determined by metabolomic profiling in non-diabetic controls (non-DM; *n* = 76) and patients with proliferative diabetic retinopathy (PDR; *n* = 102). **b**, **c** Lactate concentrations measured colorimetrically in vitreous humor (**b**) and retinal tissue (**c**) from non-DM and diabetic (DM) rats (*n* = 9 animals per group). **d**–**h** Representative immunofluorescence images of human preretinal fibrovascular membranes from patients with PDR showing glutamine synthetase (GS, green) together with GPR81 (**d**, red), α-smooth muscle actin (α-SMA; **e**, red), SM22 (**f**, red), COL1A1 (**g**, red), or glial fibrillary acidic protein (GFAP; **h**, red). Nuclei were counterstained with DAPI (blue) (*n* = 3 patients). Boxed regions are shown at higher magnification. Scale bars: **d**, 200 μm (left panels) and 20 μm (magnified panels); **e**, 50 μm (left panels) and 20 μm (magnified panels); **f**, 50 μm (left panels) and 20 μm (magnified panels); **g**, 50 μm (left panels) and 20 μm (magnified panels); **h**, 100 μm (left panels) and 20 μm (magnified panels). For two-group comparisons, statistical testing was selected according to the normality and variance criteria described in Methods (unpaired t test, with Welch’s correction where required, or Mann–Whitney U test). ns, not significant; *P < 0.05; **P < 0.01; ***P < 0.001; ****P < 0.0001

To investigate the clinical relevance of lactate accumulation in PDR, we first assessed the abundance of lactate in human vitreous samples. Liquid chromatography–mass spectrometry (LC–MS)-based untargeted metabolomic analysis revealed significantly higher vitreous lactate abundance in patients with PDR than in nondiabetic controls with macular holes (MHs), idiopathic epiretinal membranes (ERMs), or rhegmatogenous retinal detachment (RRD) (Fig. 1a, Table S1). To determine whether a similar metabolic alteration was present in experimental diabetes, a STZ-induced diabetic rat model was established. Six months after STZ injection, compared with age-matched nondiabetic control rats, diabetic rats exhibited significantly elevated lactate concentrations in both the vitreous humor and neural retina (Fig. 1b, c). These findings indicate that increased intraocular lactate is a metabolic feature shared by clinical PDR and experimental diabetes.

Because Müller glial cells span the neural retina and undergo extensive reactive remodeling during DR, we next examined whether they express molecular components involved in lactate sensing and transport. Multicolor immunofluorescence staining of human PDR fibrovascular membranes and rat retinal sections revealed partial colocalization of the lactate-responsive receptor GPR81 with the Müller cell marker glutamine synthetase (GS) (Fig. 1d, Fig. S1a). Monocarboxylate transporter 1 (MCT1), a major monocarboxylate transporter involved in lactate transport, also partially colocalized with GS in rat retinal sections (Fig. S1b). These observations indicate that Müller cells express molecular components associated with lactate sensing and transport, providing a cellular basis for their potential responsiveness to changes in lactate levels within the retinal microenvironment.

We subsequently examined the spatial relationship between glial and myofibroblast-associated markers in human PDR fibrovascular membranes. Multicolor immunofluorescence staining revealed that GS-positive and GFAP-positive glial structures partially overlapped with structures positive for the myofibroblast-associated markers α-SMA, SM22α, and COL1A1 (Fig. 1e–h). These findings demonstrate the coexistence and partial spatial overlap of glial and myofibroblast-associated features within clinical PDR fibrovascular membranes, which is consistent with the presence of glial cells that display myofibroblast-like characteristics in fibroproliferative lesions.

**Fig. 2:**
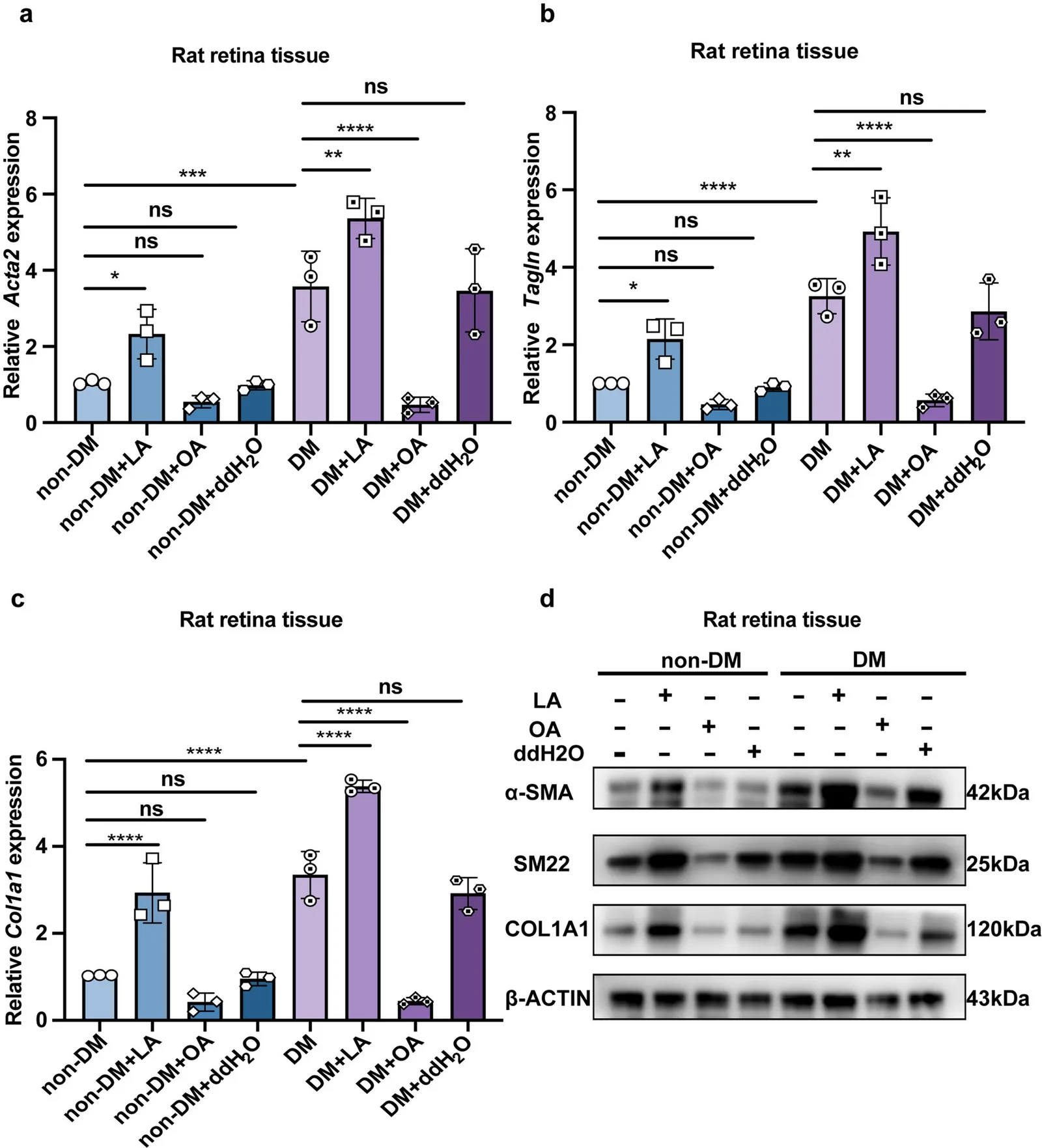
Modulation of lactate levels alters myofibroblast-associated marker expression in diabetic rat retina.** a**–**c** qPCR analysis of *Acta2* (**a**), *Tagln* (**b**), and *Col1a1* (**c**) mRNA expression in retinas from non-diabetic (non-DM) and diabetic (DM) rats following intravitreal administration of lactate (LA), oxamate (OA), or ddH2O vehicle. Nine rats were included in each group; retinas from three rats were pooled to generate one biological replicate, yielding three pooled biological replicates per group. Each plotted point represents one pooled biological replicate. **d** Representative western blots of α-SMA, SM22, and COL1A1 in retinal tissue from the same treatment groups; β-ACTIN served as the loading control. Data in (**a**–**c**) are presented as mean ± SD. For multigroup comparisons, one-way ANOVA or Kruskal–Wallis testing was used according to the distributional assumptions described in Methods, with Benjamini–Hochberg correction for multiple comparisons. ns, not significant; *P < 0.05; **P < 0.01; ***P < 0.001; ****P < 0.0001

We next examined whether pharmacological modulation of lactate-related metabolism was associated with changes in myofibroblast-associated marker expression in retinal tissue. Rats received intravitreal administration of lactate (LA) or oxamate (OA), a pyruvate analog that competitively inhibits lactate dehydrogenase activity and suppresses lactate production. Retinal analyses following intravitreal administration revealed that exogenous lactate increased the protein abundance of the myofibroblast-associated markers α-SMA, SM22α, and COL1A1. In contrast, oxamate treatment markedly reduced the protein levels of these markers, with corresponding decreasing trends in their mRNA levels (Fig. 2a–d). The opposing effects of lactate supplementation and LDH inhibition on myofibroblast-associated marker expression further support a potential link between lactate-related metabolic regulation and fibroproliferative remodeling in retinal tissue.

Taken together, these results identify increased intraocular lactate levels as a metabolic feature of both clinical PDR and experimental diabetes, demonstrate that Müller cells possess molecular components involved in lactate sensing and transport, and reveal a spatial association between glial and myofibroblast-associated features within human PDR fibrovascular membranes. Moreover, exogenous lactate and pharmacological inhibition of LDH expression had opposite effects on myofibroblast-associated marker expression in retinal tissue. These in vivo observations provide a rationale for subsequently determining whether lactate directly induces myofibroblast-like remodeling of primary Müller cells and whether inhibition of lactate production attenuates this response in vitro.

### Lactate contributes to high-glucose-induced myofibroblast-like remodeling of Müller cells

To determine whether Müller cells directly respond to lactate accumulation, primary rat retinal Müller cells were isolated and characterized by immunofluorescence staining and flow cytometry. The cells showed positive staining for Müller cell markers, including GS, CRALBP, GLAST, AQP4, and VIMENTEN, but lacked detectable expression of the microglial marker IBA1, the endothelial marker CD31, and the retinal ganglion cell marker RBPMS (Fig. S2a–c). Flow cytometric analysis further revealed that the proportions of GS-positive and CRALBP-positive cells were greater than 95% (Fig. S2d, e), confirming that the purity of the Müller cell cultures was sufficient for subsequent experiments.

**Fig. 3:**
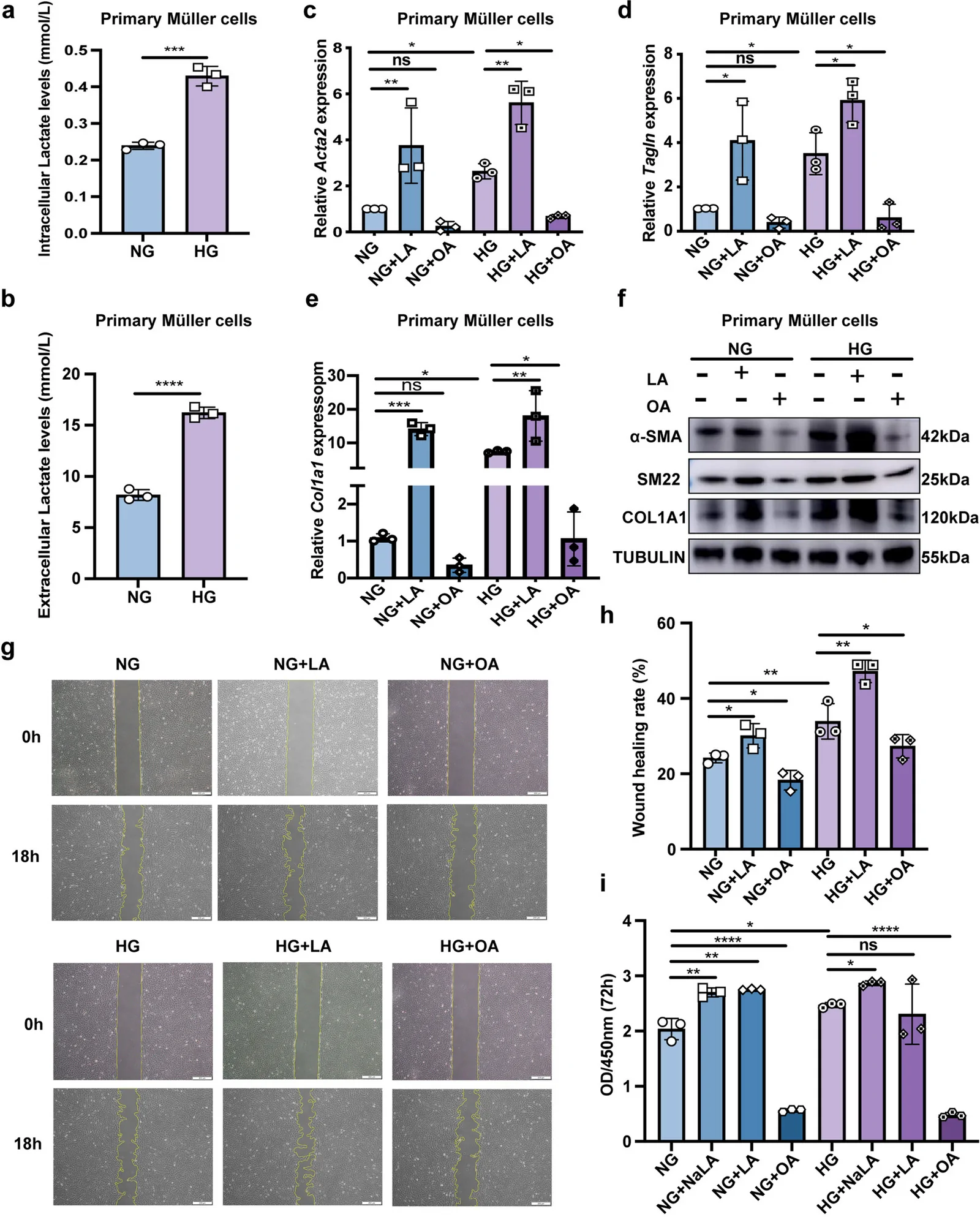
Lactate promotes myofibroblast-like activation, migration, and proliferation of Müller cells.** a**, **b** Intracellular (**a**) and extracellular (**b**) lactate concentrations in primary Müller cells cultured under normal-glucose (NG, 5.5 mM) or high-glucose (HG, 25.5 mM) conditions (*n* = 3 biologically independent cultures). **c**–**e** qPCR analysis of *Acta2* (**c**), *Tagln* (**d**), and *Col1a1* (**e**) mRNA expression in Müller cells cultured under NG or HG conditions with lactate (LA) or oxamate (OA) as indicated (*n* = 3 biologically independent cultures). **f** Representative western blots of α-SMA, SM22, and COL1A1 under the indicated conditions; tubulin served as the loading control (*n* = 3 biologically independent experiments). **g**, **h** Representative scratch-wound images at 0 and 18 h (**g**) and quantification of wound-healing rate (**h**) under the indicated conditions (*n* = 3 biologically independent experiments). Scale bars: 200 μm. **i** CCK-8 assay was measured at 450 nm after 72 h in Müller cells cultured under NG or HG conditions with LA, sodium lactate (NaLA), or OA as indicated (*n* = 3 biologically independent experiments). Data are presented as mean ± SD. Two-group or multigroup statistical tests were selected according to the normality and variance criteria described in Methods; multigroup comparisons were corrected using the Benjamini–Hochberg procedure. ns, not significant; *P < 0.05; **P < 0.01; ***P < 0.001; ****P < 0.0001

Primary Müller cells were cultured under normal-glucose (NG) or high-glucose (HG) conditions to model diabetes-associated metabolic stress. High-glucose exposure resulted in a significant increase in both intracellular and extracellular lactate levels (Fig. 3a, b). We next examined whether lactate itself could promote Müller cell remodeling. Under normal-glucose conditions, lactate supplementation increased the expression of myofibroblast-associated markers, including α-SMA, SM22α, and COL1A1, whereas the addition of lactate under high-glucose conditions further enhanced these responses (Fig. 3c–f). Conversely, inhibition of endogenous lactate production with oxamate attenuated the high glucose-induced increases in the expression of these markers. These findings indicate that lactate is capable of promoting the acquisition of myofibroblast-like molecular features in primary Müller cells.

Functional assays were subsequently performed to determine whether these molecular changes were accompanied by alterations in cellular behavior. Lactate exposure accelerated scratch-wound closure, whereas oxamate attenuated this response (Fig. 3g, h). CCK-8 assays further revealed that exogenous lactate significantly increased cellular activity under normal-glucose conditions (Fig. 3i). Under high-glucose conditions, however, the addition of lactate did not further increase the CCK-8 signal compared with that under high-glucose conditions alone. Because high-glucose exposure itself induced substantial lactate accumulation, we considered whether the addition of lactic acid might result in extracellular acidification that could partially mask the lactate-associated increase in cellular activity. To address this possibility, the cells were also treated with sodium lactate (NaLA). NaLA significantly increased CCK-8-measured cellular activity under high-glucose conditions, suggesting that the lactate-associated effect was not solely attributable to extracellular acidification. In contrast, oxamate markedly reduced cellular activity under both normal- and high-glucose conditions (Fig. 3i).

Collectively, these findings show that high-glucose exposure promotes lactate accumulation in primary Müller cells and that exogenous lactate enhances myofibroblast-associated marker expression and cellular behaviors associated with Müller cell remodeling. Conversely, pharmacological inhibition of endogenous lactate production attenuates several high-glucose-induced responses. These results support a functional contribution of lactate to high-glucose-induced myofibroblast-like remodeling of Müller cells and prompted us to investigate the molecular mechanism through which increased lactate availability is translated into sustained phenotypic changes.

### High glucose enhances MBD2 lactylation and identifies K186 as a candidate regulatory site in Müller cells

**Fig. 4:**
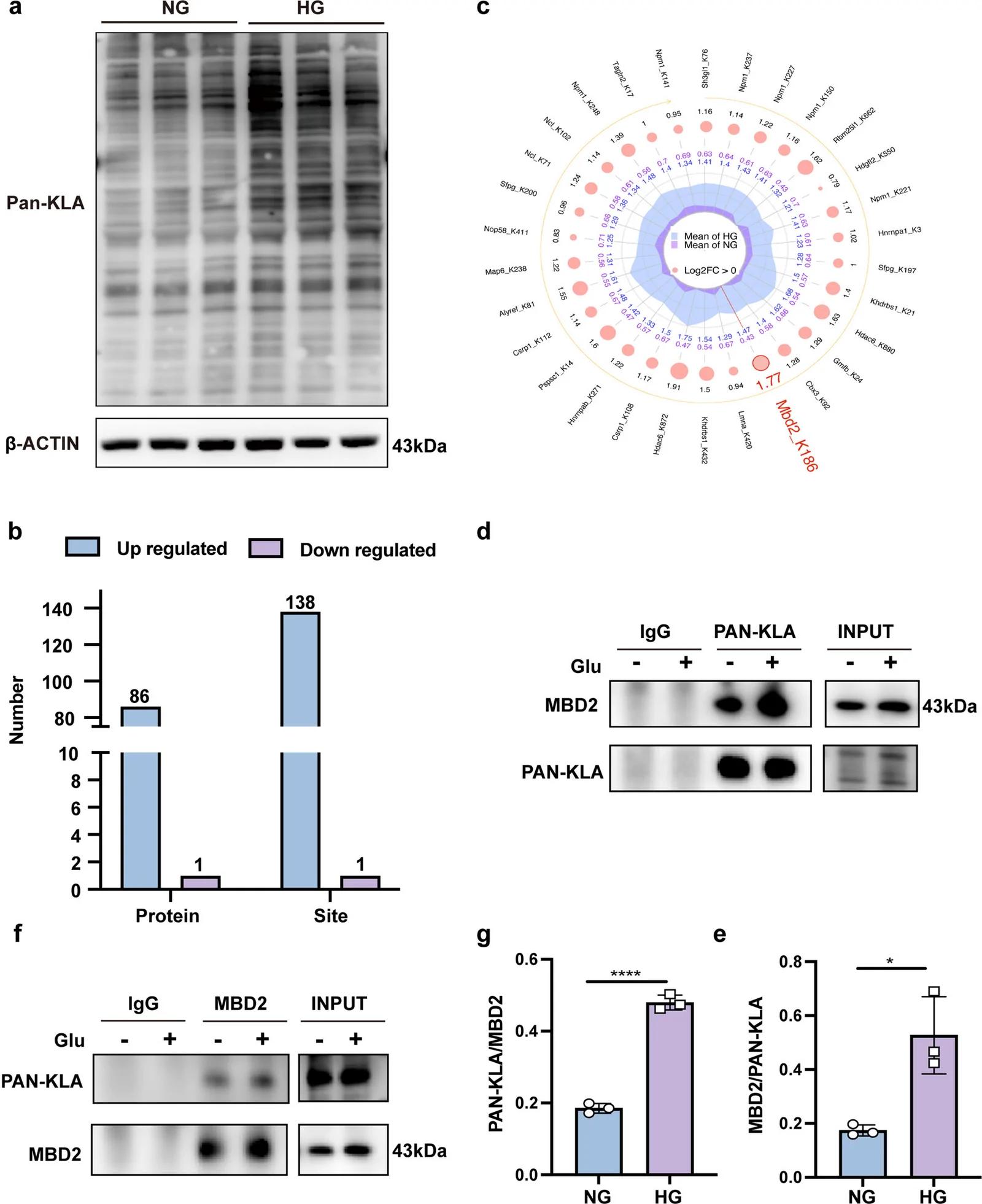
High glucose increases MBD2 lactylation and identifies K186 as a hyperlactylated site in Müller cells.** a** Representative western blot of global lysine lactylation (Pan-KLA) in primary Müller cells cultured under NG or HG conditions; β-ACTIN served as the loading control (*n* = 3 biologically independent experiments). **b** Numbers of differentially lactylated proteins and lysine sites identified by quantitative lactylome profiling under HG versus NG conditions (*n* = 3 biological replicates per condition). **c** Radar plot of the top 30 differentially lactylated proteins/sites, highlighting MBD2 K186 among sites meeting the lactylome-selection criteria (fold change > 3 and *P* < 0.05). **d**, **f** Reciprocal IP–western blot analyses of MBD2 lactylation under the indicated glucose conditions. In (**d**), lysates were immunoprecipitated with the Pan-KLA antibody and immunoblotted for MBD2; in (**f**), lysates were immunoprecipitated with the MBD2 antibody and immunoblotted for Pan-KLA. IgG controls and input samples are shown. **e**, **g** Densitometric quantification of the reciprocal IP–western blot signals shown in (**d**) and (**f**), respectively (*n* = 3 biologically independent experiments). Data in (**e**) and (**g**) are presented as mean ± SD. Two-group comparisons were analyzed according to the normality and variance criteria described in Methods. *P < 0.05; ****P < 0.0001

Because lactate can function as a signaling metabolite through lysine lactylation, we next examined whether high-glucose exposure alters protein lactylation in Müller cells. Immunoblotting with a pan-Kla antibody revealed a marked increase in global lysine lactylation following high-glucose exposure (Fig. 4a), suggesting that the high-glucose-induced increase in lactate was accompanied by increased protein lactylation.

To identify the lactylated proteins potentially involved in the response of Müller cells to high glucose, quantitative LC–MS/MS-based lactylomic profiling was performed. Comparative analysis revealed 138 significantly upregulated lactylation sites across 86 proteins under high-glucose conditions (Fig. 4b, Supplementary Material 1). Among the most strongly upregulated lactylated proteins, MBD2 was of particular interest because only one lactylation site, lysine 186 (K186), was identified on MBD2 in our dataset, and lactylation at this site was significantly increased under high-glucose conditions (FC = 3.4, *p* = 0.008) (Fig. 4c). The identification of a single site on MBD2 provided a tractable candidate for subsequent site-directed functional analysis, and MBD2 was therefore prioritized for further investigation.

Reciprocal immunoprecipitation experiments were subsequently performed to confirm MBD2 lactylation. Immunoprecipitation with a Pan-Kla antibody followed by immunoblotting for MBD2 revealed increased recovery of lactylated MBD2 under high-glucose conditions. Conversely, immunoprecipitation of MBD2 followed by Pan-Kla immunoblotting similarly demonstrated increased MBD2-associated lysine lactylation after high-glucose exposure (Fig. 4d–g). Together, these results establish MBD2 as a high-glucose-responsive lactylated protein in Müller cells and identify K186 as the principal candidate lactylation site for subsequent functional investigation.

### MBD2 K186 contributes to myofibroblast-like remodeling of Müller cells

**Fig. 5:**
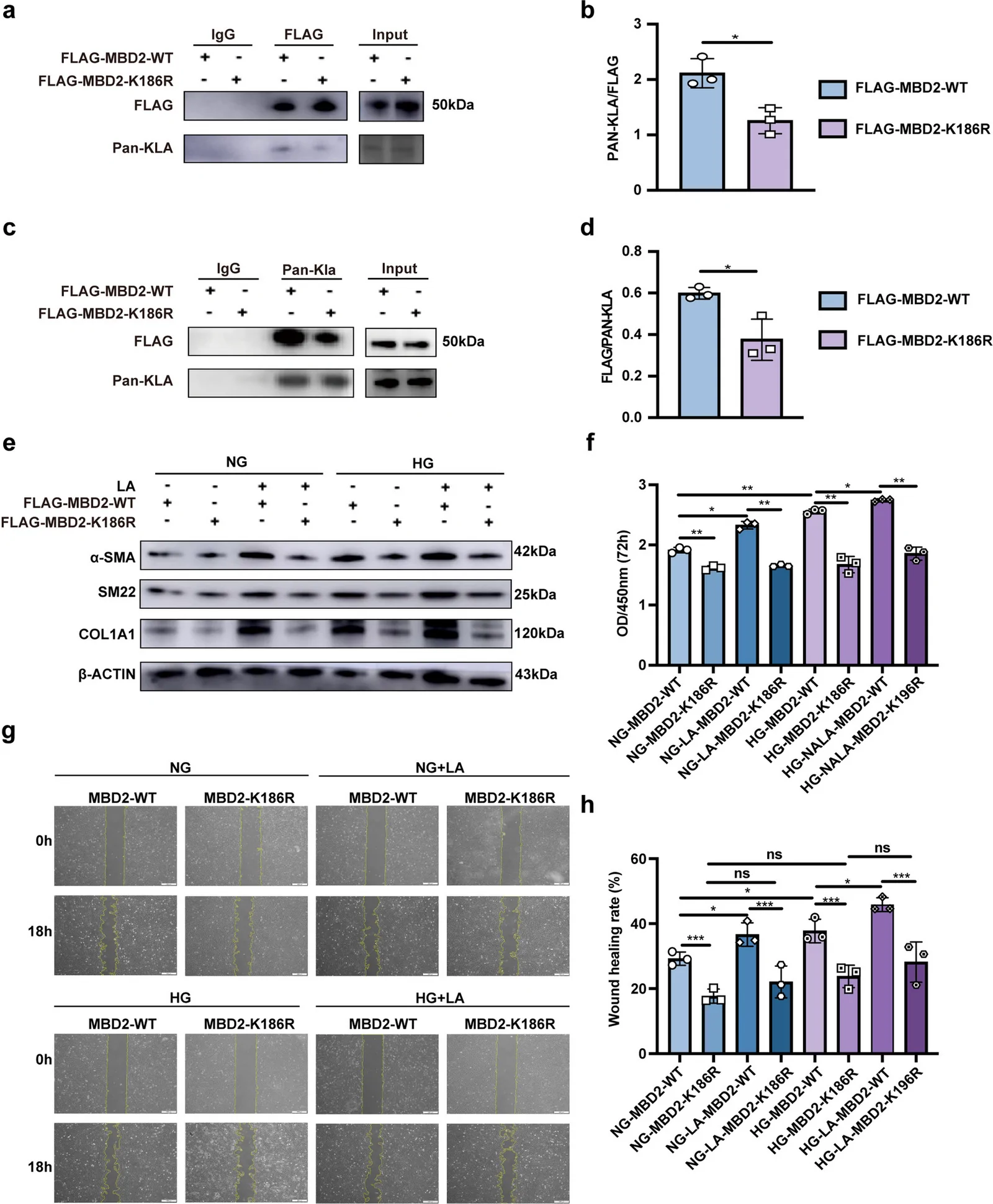
MBD2 K186 contributes to lactate-induced myofibroblast-like responses in Müller cells. **a**, **c** Reciprocal IP–Western blot analyses of FLAG-tagged wild-type MBD2 (MBD2-WT) and the K186R mutant in Müller glial cells cultured under high-glucose conditions. In (**a**), FLAG immunoprecipitates were analyzed for FLAG-MBD2 and Pan-KLA; in (**c**), Pan-KLA immunoprecipitates were analyzed for FLAG-MBD2 and Pan-KLA. IgG controls and input samples are shown (*n* = 3 biologically independent experiments). **b**, **d** Densitometric quantification of the corresponding reciprocal IP–western blot signals in (**a**) and (**c**), respectively (*n* = 3). **e** Representative western blots of α-SMA, SM22, and COL1A1 in Müller cells expressing MBD2-WT or MBD2-K186R under NG or HG conditions with or without lactate treatment; β-ACTIN served as the loading control (*n* = 3 biologically independent experiments). **f** CCK-8 assay measured at 450 nm after 72 h under the indicated MBD2-WT/MBD2-K186R and lactate/sodium-lactate treatment conditions (*n* = 3 biologically independent experiments). **g**, **h** Representative scratch-wound images at 0 and 18 h (**g**) and quantification of wound-healing rate (**h**) in Müller cells expressing MBD2-WT or MBD2-K186R under the indicated NG, HG, and lactate conditions (*n* = 3 biologically independent experiments). Scale bars: 200 μm. Data are presented as mean ± SD. Multigroup comparisons were analyzed according to the distributional assumptions described in Methods, with Benjamini–Hochberg correction for multiple comparisons. ns, not significant; *P < 0.05; **P < 0.01; ***P < 0.001

To investigate the functional relevance of the MBD2 K186 site, an MBD2 K186R substitution mutant was generated by replacing lysine 186 with arginine. Primary Müller cells expressing MBD2-WT or MBD2-K186R were subsequently subjected to lactate or high-glucose exposure. Immunoblotting confirmed the expression of both MBD2-WT and MBD2-K186R following viral transduction (Fig. S3a). Reciprocal immunoprecipitation of FLAG-tagged MBD2 and lactylated proteins from high-glucose-cultured Müller glial cells using anti-FLAG and pan-Kla antibodies, respectively, followed by immunoblotting with reciprocal antibodies, revealed that compared with MBD2-WT, the K186R substitution markedly reduced MBD2 lactylation after normalization to the corresponding immunoprecipitated protein levels (Fig. 5a–d), suggesting that K186 is a major determinant of MBD2 lactylation.

We next examined whether the K186R substitution affects the myofibroblast-like remodeling of Müller cells. Compared with MBD2-WT-expressing cells, MBD2-K186R-expressing cells showed attenuated induction of the myofibroblast-associated markers α-SMA, SM22α, and COL1A1 following lactate or high-glucose exposure (Fig. 5e). K186R substitution also attenuated the lactate-induced increases in CCK-8-induced cellular activity and scratch-wound closure (Fig. 5f–h). Together, these findings demonstrate that K186 is functionally important for MBD2 lactylation and support a role for K186-dependent MBD2 regulation in lactate- and high-glucose-associated myofibroblast-like remodeling of Müller cells.

### YAP1 contributes to MBD2 K186-dependent Müller cell remodeling

**Fig. 6:**
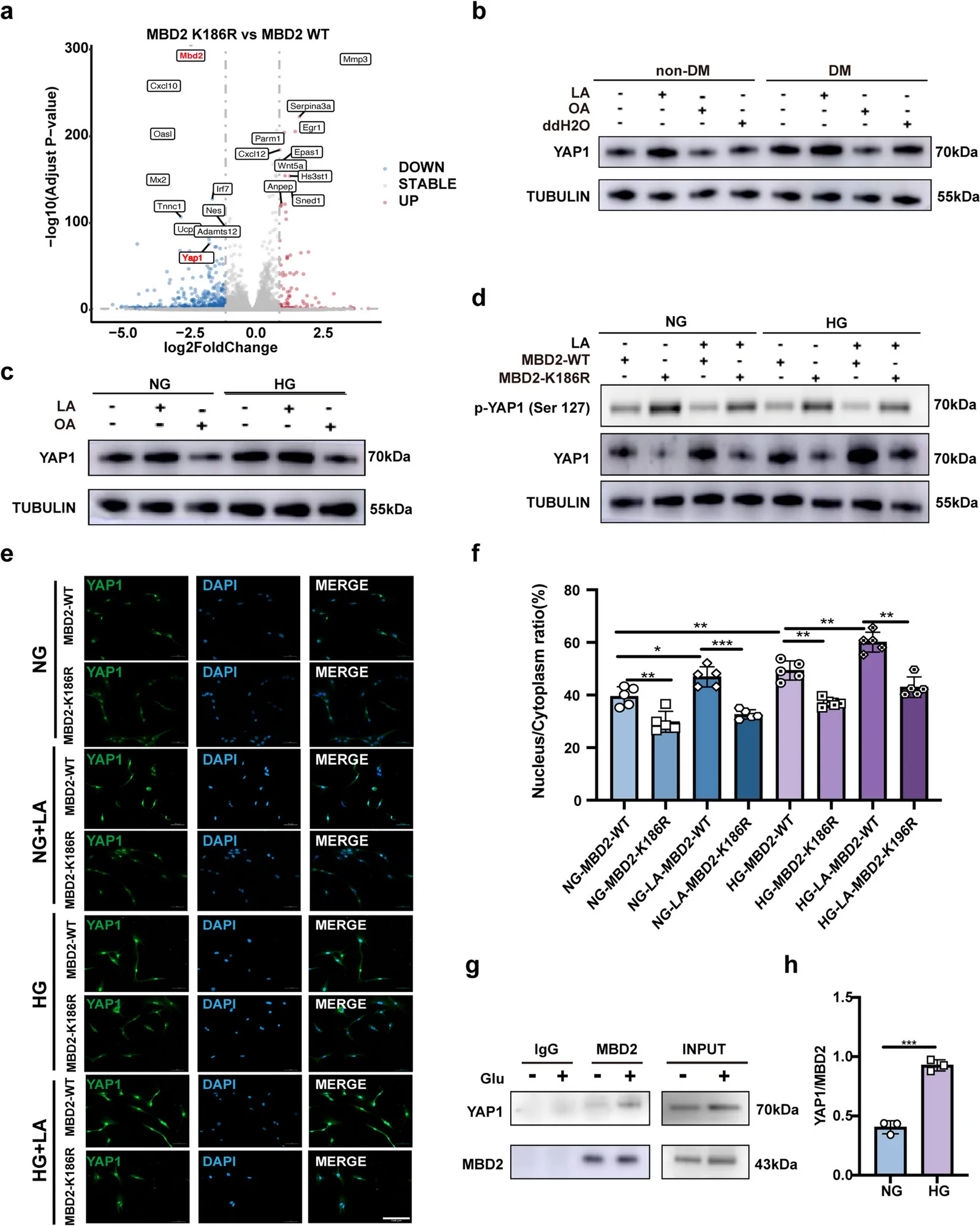
MBD2 K186-dependent regulation modulates YAP1 expression and signaling in Müller cells.** a** Volcano plot of differential gene expression in MBD2-K186R versus MBD2-WT Müller cells. MBD2 and YAP1 are highlighted among the downregulated genes; differential-expression thresholds were log2 fold change < − 1.5 and adjusted *P* < 0.05. **b** Representative western blot of YAP1 in retinal tissues from non-DM and DM rats after intravitreal administration of lactate (LA), oxamate (OA), or ddH2O vehicle; TUBULIN served as the loading control (*n* = 9 animals per group). **c** Representative western blot of YAP1 in primary Müller cells cultured under NG or HG conditions with LA or OA; TUBULIN served as the loading control (*n* = 3 biologically independent experiments). **d** Representative western blots of YAP1 phosphorylated at Ser127 [p-YAP1(Ser127)] and total YAP1 in Müller cells expressing MBD2-WT or MBD2-K186R under NG or HG conditions with or without lactate treatment; TUBULIN served as the loading control (*n* = 3 biologically independent experiments). **e**, **f** Representative immunofluorescence images of YAP1 subcellular localization (**e**) and quantification of nuclear YAP1 fluorescence (**f**) in Müller cells expressing MBD2-WT or MBD2-K186R under the indicated NG, HG, and lactate conditions (*n* = 5 biologically independent experiments). Scale bars: 50 μm. **g**, **h** Co-immunoprecipitation analysis of the MBD2–YAP1 interaction under NG and HG conditions. **g**, MBD2 immunoprecipitates were immunoblotted for YAP1 and MBD2; IgG controls and input samples are shown. **h**, Quantification of co-immunoprecipitated YAP1 normalized to immunoprecipitated MBD2 (*n* = 3 biologically independent experiments). Data are presented as mean ± SD. Multigroup comparisons were analyzed according to the distributional assumptions described in Methods, with Benjamini–Hochberg correction for multiple comparisons. ns, not significant; *P < 0.05; **P < 0.01; ***P < 0.001

To identify downstream pathways associated with MBD2 K186-dependent Müller cell remodeling, RNA sequencing was performed in Müller cells expressing MBD2-WT or MBD2-K186R under high-glucose conditions. A total of 627 differentially expressed genes were identified, including 104 upregulated genes and 523 downregulated genes, in MBD2-K186R-expressing cells compared with MBD2-WT-expressing cells (Supplementary Material 2). *Mbd2* expression was reduced in the MBD2-K186R group, which is consistent with the lower MBD2 protein abundance detected by immunoblotting (Fig. 6a, Fig. S3a), indicating that the K186R substitution was accompanied by changes in MBD2 abundance at both the transcript and protein levels.

Among the differentially expressed genes, *Yap1* was significantly downregulated in MBD2-K186R-expressing Müller cells (Fig. 6a). YAP1 is a key downstream effector of the Hippo signaling pathway and has been implicated in fibrotic remodeling and myofibroblast-like phenotypic transitions. On the basis of its differential expression and established role in cellular remodeling, YAP1 was selected for further investigation as a candidate downstream mediator of MBD2 K186-dependent regulation.

We next examined YAP1 expression under diabetic and lactate-associated conditions. Compared with that in nondiabetic control retinal tissue, the protein abundance of YAP1 in diabetic retinal tissue was increased. Intravitreal lactate administration further increased retinal YAP1 abundance, whereas oxamate treatment reduced YAP1 levels (Fig. 6b). Consistent with these in vivo findings, high-glucose exposure increased the abundance of YAP1 in Müller cells, and this effect was further enhanced by lactate supplementation. In contrast, oxamate reduced the abundance of YAP1 under both normal- and high-glucose conditions and attenuated the lactate-associated increase in YAP1 expression (Fig. 6c).

Because YAP1 activity is closely associated with its phosphorylation status and subcellular localization, we further examined the phosphorylation and nuclear accumulation of YAP1 at Ser127. High-glucose exposure decreased relative YAP1 Ser127 phosphorylation and promoted YAP1 nuclear accumulation, and lactate supplementation further enhanced these changes. In contrast, MBD2-K186R expression increased relative YAP1 Ser127 phosphorylation and reduced nuclear YAP1 accumulation under both normal- and high-glucose conditions but attenuated the response to lactate supplementation (Fig. 6d–f). These findings indicate that MBD2 K186-dependent regulation is associated with changes in both the abundance and activation state of YAP1.

Coimmunoprecipitation further revealed that the association between MBD2 and YAP1 was significantly stronger under high-glucose conditions than under normal-glucose conditions, as determined by normalization of coimmunoprecipitated YAP1 to immunoprecipitated MBD2 (Fig. 6g, h). These findings further support a molecular association between MBD2 and YAP1 that is strengthened in response to high-glucose stimulation.

**Fig. 7:**
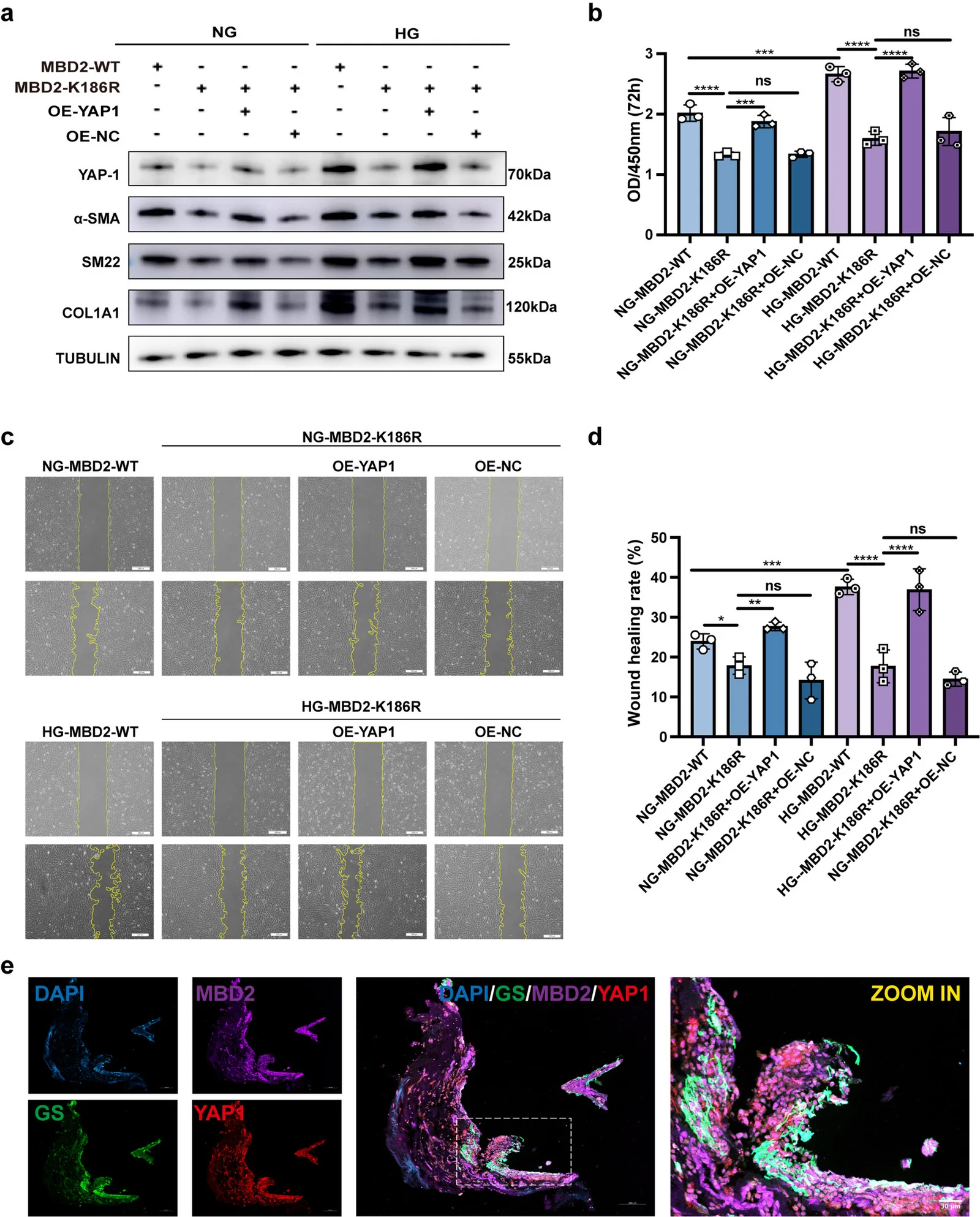
YAP1 mediates MBD2 K186-dependent myofibroblast-like remodeling of Müller cells.** a** Representative western blots of YAP1, α-SMA, SM22, and COL1A1 in Müller cells expressing MBD2-WT or MBD2-K186R, with YAP1 overexpression (OE-YAP1) or overexpression control (OE-NC) where indicated, under NG or HG conditions; TUBULIN served as the loading control (*n* = 3 biologically independent experiments). **b** CCK-8 assay measured at 450 nm after 72 h under the indicated conditions (*n* = 3 biologically independent experiments). **c**, **d** Representative scratch-wound images (**c**) and quantification of wound-healing rate (**d**) in MBD2-WT and MBD2-K186R Müller cells with OE-YAP1 or OE-NC under NG or HG conditions (*n* = 3 biologically independent experiments). Scale bars: 200 μm. **e** Representative immunofluorescence images of human PDR preretinal fibrovascular membranes stained for glutamine synthetase (GS, green), MBD2 (magenta), and YAP1 (red), with DAPI nuclear counterstaining (blue) (*n* = 3 patients). The boxed/selected region is shown at higher magnification; scale bars, 100 μm (overview) and 30 μm (magnified image). Data in (**b**) and **d** are presented as mean ± SD. Multigroup comparisons were analyzed according to the distributional assumptions described in Methods, with Benjamini–Hochberg correction for multiple comparisons. ns, not significant; *P < 0.05; **P < 0.01; ***P < 0.001; ****P < 0.0001

To determine whether YAP1 functionally contributes to MBD2 K186-dependent Müller cell remodeling, YAP1 was overexpressed in MBD2-K186R-expressing Müller cells (Fig. S3b). YAP1 overexpression restored the expression of myofibroblast-associated proteins and partially reversed the decrease in CCK-8-induced cellular activity and scratch-wound closure that were attenuated by MBD2-K186R expression (Fig. 7a–d). These rescue experiments support a functional contribution of YAP1 to the remodeling phenotype associated with MBD2 K186-dependent regulation.

Finally, triple immunofluorescence staining of human PDR fibrovascular membranes revealed spatial overlap of MBD2 and YAP1 within GS-positive glial structures (Fig. 7e). Although this spatial co-occurrence does not establish direct molecular regulation in human tissues, it provides clinical tissue evidence for the coexistence of MBD2- and YAP1-associated features in Müller glial structures within PDR lesions.

Collectively, these findings identify YAP1 as a functionally relevant downstream mediator that contributes, at least in part, to MBD2 K186-dependent myofibroblast-like remodeling of Müller cells.

**Fig. 8:**
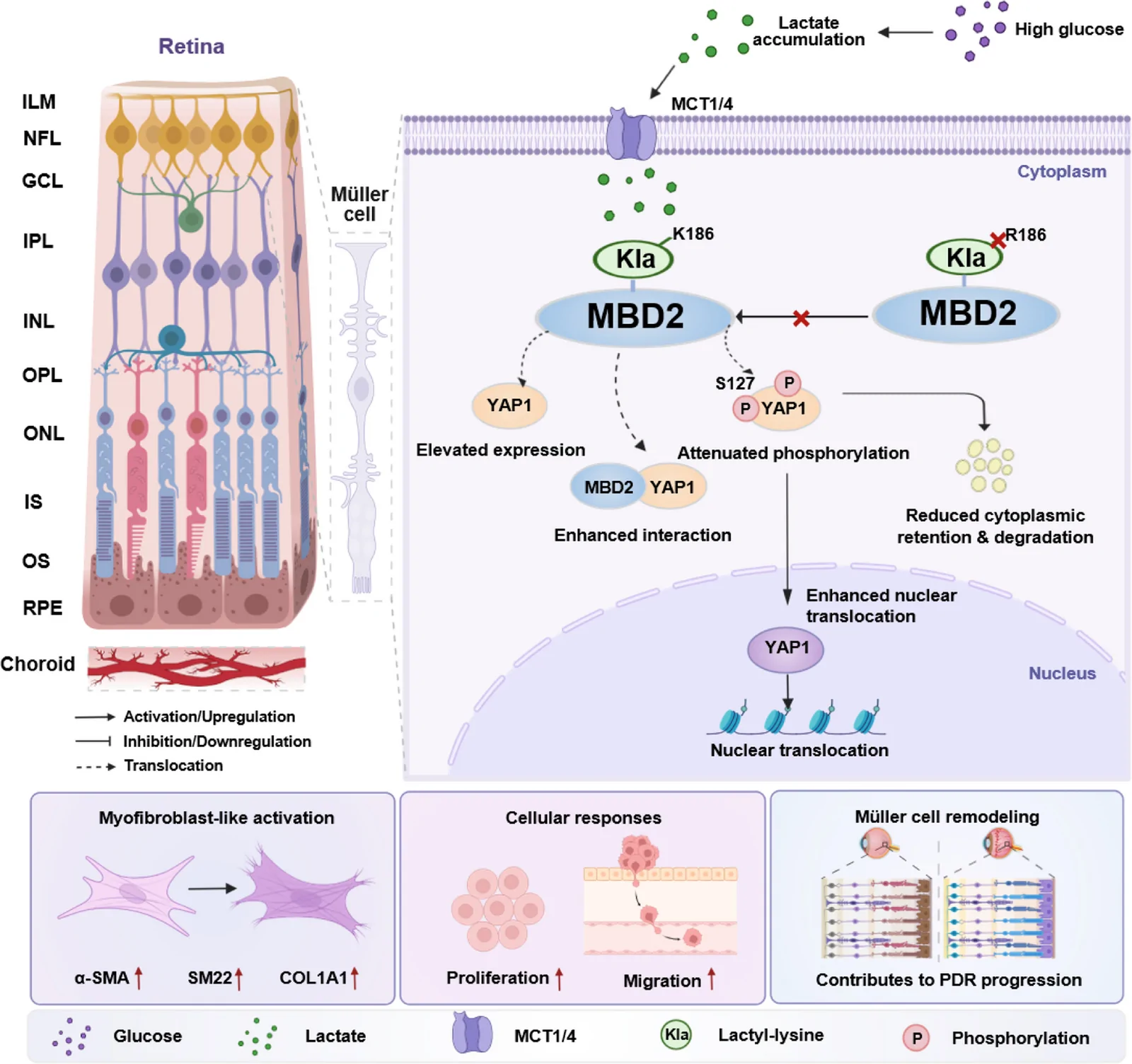
Proposed working model of the lactate–MBD2–YAP1 axis in Müller cell myofibroblast-like remodeling. Under diabetic/high-glucose conditions, lactate accumulation promotes MBD2 K186 lactylation in Müller cells, which is associated with enhanced MBD2–YAP1 interaction, reduced YAP1 Ser127 phosphorylation, and increased YAP1 nuclear accumulation. Disruption of MBD2 K186 lactylation by the K186R substitution reverses these changes and limits YAP1 nuclear translocation. Activation of this lactate–MBD2–YAP1 regulatory axis is accompanied by increased expression of myofibroblast-associated markers, including α-SMA, SM22α, and COL1A1, together with enhanced cellular remodeling. Collectively, these findings support a model in which MBD2 K186 lactylation links lactate accumulation to YAP1-dependent Müller cell myofibroblast-like remodeling in diabetic retinopathy. The figure was created using BioRender [License ID: YJ2A80U1KG]

On the basis of these findings, we propose a working model in which high-glucose-associated lactate accumulation promotes MBD2 K186 lactylation and MBD2 K186-dependent YAP1 regulation, thereby contributing to myofibroblast-like remodeling of Müller cells in PDR (Fig. 8).

## Discussion

This study supports a lactate-responsive molecular mechanism linking diabetes-associated metabolic stress to myofibroblast-like phenotypic remodeling of retinal Müller cells. Elevated lactate increased the expression of contractile and extracellular matrix-associated proteins and enhanced Müller cell proliferation and migration. Mechanistically, MBD2 was identified as a nonhistone lactylated protein, and K186 was shown to be functionally involved in the regulation of YAP1-associated cellular responses. Importantly, MBD2 K186R attenuated YAP1 activation and Müller cell remodeling, whereas YAP1 overexpression partially reversed these phenotypic changes, functionally positioning YAP1 downstream of K186-dependent MBD2 regulation. These findings extend the biological significance of lactate beyond its conventional role as a glycolytic metabolite and reveal a previously unrecognized connection among metabolic alteration, protein lactylation, and Müller cell remodeling.

Lactate accumulation is increasingly recognized as a characteristic feature of metabolically stressed tissues. In the diabetic retina, enhanced glycolytic activity, retinal hypoxia, and impaired metabolic homeostasis may contribute to increased lactate accumulation, which is consistent with vitreous metabolomic findings from patients with PDR and observations in experimental diabetic models [13, 32, 33]. Beyond its role as an energy metabolite, lactate can regulate cellular behavior through extracellular GPR81 signaling, monocarboxylate transporter-mediated transport, redox regulation, and lysine lactylation [14–16, 19, 22]. Under our experimental conditions, elevated lactate levels were sufficient to induce myofibroblast-like remodeling of Müller cells, as indicated by increased α-SMA, SM22α, and COL1A1 expression together with enhanced proliferative and migratory activity. These observations are consistent with previous studies showing that lactate promotes fibrogenic responses in pulmonary, hepatic, and renal tissues [17, 18, 20, 21, 34]. Nevertheless, lactate should not be considered the sole driver of diabetic Müller cell dysfunction. Hyperglycemia, oxidative stress, inflammatory cytokines, hypoxia, and extracellular matrix alterations are likely to act in parallel or converge on overlapping signaling networks that collectively affect Müller cell behavior in the diabetic retina.

The phenotypic alterations observed in the present study should also be interpreted in the context of Müller cell plasticity. Müller cells are the principal retinal macroglia and play essential roles in maintaining metabolic, neuronal, and vascular homeostasis. Under persistent diabetic stress, Müller cells undergo reactive gliosis and can acquire contractile and extracellular matrix-producing features that may contribute to fibrovascular remodeling [8–12]. Accordingly, the increased expression of α-SMA, SM22α, and COL1A1 in our study supports the acquisition of myofibroblast-like characteristics rather than the establishment of a definitive glial-to-mesenchymal lineage conversion. Given the heterogeneous cellular composition of fibrovascular membranes, Müller cells should therefore be considered important potential contributors, but not necessarily the exclusive cellular source, to retinal fibrotic remodeling.

The identification of MBD2 as a lactate-responsive lactylated protein provides a potential mechanistic bridge between metabolic alterations and chromatin-associated transcriptional regulation. MBD2 is classically recognized as a methyl-CpG-binding protein involved in chromatin organization and transcriptional control [23, 35, 36]. Emerging studies have further implicated MBD2 in diabetic retinal injury and fibrotic disorders through its effects on macrophage polarization and fibroblast activation [24–26, 37]. Our findings extend these observations by identifying MBD2 as a nonhistone lactylated protein and implicating K186 in lactate-dependent Müller cell remodeling. Notably, however, the K186R substitution reduced not only MBD2 lactylation but also total MBD2 abundance. Thus, the effects attributable specifically to the loss of K186 lactylation cannot be completely distinguished from those caused by altered MBD2 stability or abundance. Endogenous site-specific knock-in models, together with protein turnover and degradation analyses, will be important for resolving these possibilities.

Our findings further implicate YAP1 as a downstream mediator of K186-dependent MBD2 regulation. YAP1 is a central effector of the Hippo pathway that integrates biochemical and mechanical signals to regulate cell proliferation, extracellular matrix remodeling, and tissue fibrosis [27, 30, 31, 38–41]. YAP1 activation has also been reported in reactive Müller cells and in experimental retinal fibrosis [28, 29]. Notably, our results suggest that the relationship between MBD2 and YAP1 may involve more than a single regulatory mechanism. RNA-seq analysis revealed reduced YAP1 expression in cells expressing MBD2 K186R, suggesting that K186-dependent MBD2 regulation may influence YAP1 at the transcriptional or expression level. In parallel, coimmunoprecipitation demonstrated enhanced MBD2–YAP1 association under high-glucose conditions, suggesting the possibility of an additional protein-association-dependent mechanism. However, whether this interaction is directly regulated by K186 lactylation remains to be established. At the functional level, MBD2 K186R attenuated YAP1 activation and myofibroblast-like changes, whereas YAP1 overexpression partially reversed these effects. The results of the rescue experiment therefore provide functional evidence that YAP1 operates downstream of K186-dependent MBD2 regulation, whereas the RNA-seq and coimmunoprecipitation findings suggest that the regulation of YAP1 may occur at multiple levels, potentially involving its expression, association with MBD2, and activation state. Defining the relative contribution and mechanistic interplay of these regulatory layers will require further investigation.

The present findings also have potential translational implications. Current treatment strategies for PDR primarily target pathological neovascularization, whereas fibrovascular remodeling and subsequent tractional complications remain difficult to control. The lactate-responsive MBD2–YAP1 regulatory relationship identified here provides a potential mechanism through which diabetes-associated metabolic disturbance may be translated into pathological Müller cell remodeling. Nevertheless, both MBD2 and YAP1 participate in fundamental physiological processes, making global inhibition of either molecule potentially undesirable. If the upstream enzymatic machinery or molecular interactions governing MBD2 K186 lactylation can be defined, more selective modulation of this regulatory process may ultimately offer a more targeted therapeutic strategy than broad inhibition of MBD2 or YAP1 signaling.

Several limitations should be acknowledged. First, although the purity of the Müller cells exceeded 90%, minor contamination by other retinal cell types cannot be completely excluded. Additional marker-based cell sorting or single-cell analyses would further strengthen the attribution of the observed responses to Müller cells. Second, the present study lacks in vivo lineage-tracing evidence and therefore cannot establish whether Müller cells undergo stable phenotypic conversion or directly contribute to fibrovascular membranes in vivo. Inducible Müller cell-specific reporter models combined with single-cell transcriptomic analysis may help address this question. Third, because the K186R substitution altered both MBD2 lactylation and total protein abundance, site-specific endogenous editing and protein stability analyses are needed to distinguish modification-dependent effects from changes in MBD2 turnover. Fourth, although our data functionally position YAP1 downstream of K186-dependent MBD2 regulation, the molecular mechanisms linking these events remain incompletely defined. Future studies combining chromatin-based approaches, transcriptional analyses, protein-interaction mapping, and subcellular localization assays will be needed to determine whether MBD2 regulates YAP1 transcription, protein association, activation, or a combination of these processes. Finally, the absence of in vivo validation limits direct extrapolation of the present findings to PDR. This issue is further complicated by the lack of an established animal model that fully recapitulates the fibrovascular proliferation characteristic of human PDR, emphasizing the need for appropriate experimental models to validate the proposed mechanism in vivo.

In conclusion, this study revealed a previously unrecognized lactate-responsive MBD2–YAP1 regulatory pathway associated with myofibroblast-like remodeling of retinal Müller cells. These data functionally implicate MBD2 K186 in the regulation of YAP1 and suggest that protein lactylation may represent an important molecular interface linking diabetes-associated metabolic stress to retinal fibrovascular remodeling. Although the precise molecular consequences of MBD2 K186 lactylation require further clarification, this regulatory relationship provides a mechanistic framework for understanding the metabolic control of Müller cell plasticity and a potential direction for future therapeutic investigations in PDR.

## Materials and methods

### Human vitreous specimens

In 2022, we enrolled 178 patients at Shanghai General Hospital, comprising 102 with newly diagnosed PDR and 76 non‑diabetic patients undergoing vitrectomy for ERM, RRD or MH. The study adhered to the Declaration of Helsinki and obtained approval from the Ethics committee of Shanghai General Hospital (No.2013KY023). Written informed consent was obtained from all participants. PDR was defined by iris/retinal neovascularization or imaging evidence of vitreous/preretinal hemorrhage, as confirmed by two retinal specialists (KL and LS). A 0.3 mL sample of undiluted vitreous was collected before vitrectomy, placed in cryotubes, flash-frozen with liquid nitrogen, and stored at −80 °C in the hospital's Ophthalmic Biobank. The processing details are provided in the Supplementary Material 3.

### STZ-induced diabetic rat model

Adult male (more stable hormone levels) Sprague–Dawley rats (250‑300 g) were obtained from the Shanghai Laboratory Animal Center, China. Healthy male SD rats were randomized into two groups: diabetes mellitus (DM) and non-DM. A 10 mg/mL solution of Streptozotocin (STZ) was prepared by dissolving the powder in 0.1 mol/L sodium citrate buffer and sterilizing it using a 0.22 μm bacterial filter. STZ was administered intraperitoneally at a dose of 45 mg/kg. Diabetes was verified in rats with blood glucose levels exceeding 16.7 mmol/L, measured weekly via tail vein using a Roche precision glucometer following STZ injection. Six months post-STZ injection, all rats were euthanized. The Ethics Committee of Shanghai General Hospital approved the study (No. 2023AW012).

### Primary Müller cell isolation and culture

Prepare serum-free Dulbecco Modified Eagle Medium (DMEM, Gibco 11,885,084), penicillin/streptomycin (P/S, Yeasen, 60162ES76), 0.25% (v/v) trypsin (Gibco 25,200,056), fetal bovine serum (FBS, Gibco A5256701) and 1 × phosphate buffered saline (PBS, Servicebio G4202-500ML). P/S was supplemented at a final concentration of 1% (v/v) into serum-free DMEM, 1 × PBS and DMEM containing 10% FBS (v/v), to prepare Müller cell eye solution (serum-free DMEM supplemented with 1% P/S), P/S-containing 1 × PBS, and complete primary Müller cell growth medium (DMEM supplemented with 10% FBS and 1% P/S), respectively. 10 to 20 day-old specific pathogen-free (SPF) Sprague–Dawley (SD) rats (purchased from Changzhou Cavens Laboratory Animal Co., Ltd.) were disinfected by immersion in 75% (v/v) ethanol for 5 min. Eyelids and fascia were incised under aseptic conditions, and eyeballs were enucleated. The isolated eyeballs were rinsed no less than three times with P/S-containing 1 × PBS to fully remove blood and impurities. Afterwards, eyeballs were immersed in Müller cell eye solution and incubated overnight at 37 °C with 5% CO_2_. After overnight incubation, the biosafety cabinet was sterilized by ultraviolet irradiation for 30 min. The eyeballs were taken out, and the supernatant DMEM was discarded. Tissues were rinsed once with P/S-containing 1 × PBS, then transferred into 2 mL Eppendorf (EP) tubes filled with 0.25% trypsin for digestion at 37 °C for 1.5–2 h. Digestion was terminated by transferring eyeballs into a 60 mm culture dish preloaded with approximately 5 mL complete primary Müller cell growth medium. Eyeballs were circumferentially cut along the ora serrata to remove anterior ocular segments and vitreous body. Retinas were gently separated from sclera and repeatedly washed with P/S-containing 1 × PBS to eliminate adherent retinal pigment epithelial cells. Isolated retinal tissues were transferred into fresh P/S-containing 1 × PBS, minced with ophthalmic scissors and collected into 15 mL centrifuge tubes. Samples were centrifuged at 1000 rpm for 5 min, and the supernatant was discarded. The cell pellet was gently rinsed with buffer solution and resuspended in complete primary Müller cell growth medium via mild pipetting. The cell suspension was seeded into 25 cm^2^ culture flasks at a density of 2 × 10^6^cells per cm^2^, and cultured at 37 °C with 5% CO_2_. Cell adhesion was observed daily from the next day. Culture medium was refreshed every 2–3 days. Cells were passaged when cell confluence reached no less than 80%. Cells at passages 2 to 4 were adopted for subsequent experiments.

### LC‑MS metabolomics

Metabolomic profiling was performed using an Ultimate 3000 UHPLC system coupled to a Q-Exactive mass spectrometer (Thermo Fisher Scientific, USA). Metabolites were separated on an HSS T3 column and analyzed by electrospray ionization mass spectrometry in positive/negative ion switching mode with full-scan acquisition over an m/zrange of 200–2000. Detailed chromatographic gradients and mass spectrometric parameters are provided in the Supplementary Material 3.

### Intravitreal modulation of lactate

To modulate retinal lactate, rats were anesthetized with 2% isoflurane and received intravitreal injections (5 μL) of either sodium lactate (10 mmol/L; buffered to pH ≈ 7.3, selected to approximate physiologically relevant lactate exposure) or sterile phosphate-buffered saline (PBS, as control) using a Hamilton syringe fitted with ~32-gauge glass capillaries. The final intraocular lactate concentration was within pathophysiologic ranges (≈5–20 mmol/L). In separate experiments, 20 mmol/L oxamate was injected to reduce lactate production. Animals were euthanized 24 h after injection.

### Lactate quantification

Age-matched STZ-induced diabetic and control (room air) rats were euthanized. Their vitreous and retinas were snap-frozen in liquid nitrogen and then stored at −80 °C refrigerator. For lactate quantification, 50 mg retinal fragments or 5 μL vitreous samples were homogenized and analyzed using a test kit (Nanjing Jiancheng, China). Readings were got at 530 nm by Multiskan SkyHigh microplate reader (Thermo Fisher Scientific, USA), with results converted to concentration (mmol/L).

### Lentiviral transduction and transient plasmid transfection

Recombinant lentiviruses encoding wild-type MBD2 (pcSLenti-EF1-EGFP-P2A-Puro-CMV-Mbd2-3 × FLAG-WPRE) and the MBD2 K186R mutant (pcSLenti-EF1-EGFP-P2A-Puro-CMV-Mbd2(K186R) − 3 × FLAG-WPRE), together with the YAP1 expression plasmid (pCDNA3.1-CMV-Yap1-hGHpolyA-EF1α-EGFP) and the corresponding empty vector (pCDNA3.1-CMV-hGHpolyA-EF1α-EGFP) were purchased from OBiO Technology (Shanghai) Corp., Ltd. (Shanghai, China). The lentiviral vectors used for MBD2 and MBD2-K186R overexpression contained both EGFP and puromycin-resistance genes. For lentiviral transduction, cells were infected with the corresponding MBD2 or MBD2-K186R lentivirus at a multiplicity of infection (MOI) of 100. Successful transduction was initially evaluated by monitoring EGFP fluorescence under a fluorescence microscope. Once green fluorescence was evident, the transduced cells were selected with 4 μg/mL puromycin for 48 h and subsequently collected for downstream experiments. For transient YAP1 overexpression, cells were seeded one day before transfection and transfected when they reached approximately 50–80% confluence. Transfection was performed using PEI Prime™ linear polyethylenimine (Sigma-Aldrich, St. Louis, MO, USA; Cat. No. 919012). A sterile PEI stock solution at 1 mg/mL was used. The YAP1 plasmid and PEI were separately diluted in serum-free DMEM at amounts of 1.0 μg plasmid DNA and 3.0 μg PEI per 10^6^ cells, corresponding to a DNA-to-PEI mass ratio of 1∶3. The diluted plasmid DNA and PEI solutions were mixed and incubated for 10 min at room temperature to allow the formation of DNA–PEI complexes. The complexes were then added dropwise to the cells. At 48 h after transfection, the cells were collected for subsequent analyses. The overexpression efficiencies of MBD2, MBD2-K186R, and YAP1 were further verified by Western blotting. The representative Western blot results (Fig. S3) and corresponding vector maps (Fig. S4) are provided in the Supplementary Material 3.

### Real-time quantitative PCR (qRT-PCR)

Total RNA was extracted using TRIzol reagent and reverse-transcribed into cDNA using PrimeScript RT Master Mix. Quantitative PCR was performed using SYBR Premix Ex Taq on an ABI 7500HT system, with *β-Actin* used as the internal reference. Detailed procedures and primer sequences are provided in the Supplementary Material 3.

### Western blotting and immunoprecipitation

Proteins from retinal tissues and cultured cells were extracted using Western and IP lysis buffer, and protein concentrations were determined by BCA assay. For Western blotting, equal amounts of protein were separated by SDS-PAGE, transferred to nitrocellulose membranes, and immunoblotted with the indicated antibodies. Band intensities were quantified using ImageJ and normalized to the corresponding loading controls and untreated controls, where applicable. For immunoprecipitation, cell lysates were incubated with the indicated antibodies or corresponding control IgG, followed by capture of the immune complexes using Protein A/G agarose beads. The immunoprecipitated proteins were subsequently analyzed by Western blotting. Detailed procedures are provided in the Supplementary Material 3.

### Flow cytometry

Cultured primary rat retinal Müller cells were washed twice with PBS, detached with 0.25% trypsin, and centrifuged at 300 × g for 5 min. The cells were resuspended in ice-cold PBS and adjusted to 1 × 10^6^ cells per tube. After fixation and permeabilization, cells were blocked for 15 min at room temperature to reduce nonspecific antibody binding. Separate cell aliquots were incubated with unconjugated primary antibodies against CRALBP or GS for 40 min at 4 °C. After washing, the cells were incubated with the corresponding APC- or FITC-conjugated secondary antibodies for 30 min at 4 °C in the dark. Cells were washed, resuspended in PBS, and analyzed using a BD FACSAria II flow cytometer. Data were processed using FlowJo software version 10.8, and the percentages of CRALBP-positive and GS-positive cells were determined separately. Antibody information are provided in supplementary Material 3.

### Imaging modality and parameters

Fixed tissues and cells were imaged using a Leica SP8 confocal microscope (DMi8 inverted stand) equipped with a HC PL APO CS2 40 × oil-immersion objective (NA 1.30, WD 0.24 mm). Fluorescence was sequentially excited using diode lasers at 405 nm (DAPI), 488 nm (FITC/GFP), 552 nm (TRITC), and 638 nm (Cy5), with emission detected at 410–480 nm, 500–550 nm, 570–620 nm, and 640–750 nm, respectively. Z-stack images were acquired with a step size of 0.5 µm covering the entire cell thickness (10–15 optical sections per field). All image acquisition was performed using Leica LAS X software (version 3.5.7), and images were saved in.lif format.

### CCK-8 assay

Cells were seeded in 96‑well plates (5,000 cells/well; five replicate wells/group). After treatments, 10 μL CCK‑8 reagent (Yeasen Biotechnology, China) was added per well and incubated for 2 h at 37 °C; absorbance at 450 nm was measured (Thermo Fisher Scientific).

### Scratch-wound assay

Primary Müller cells were seeded in 6‑well plates at a density of 2 × 10^5^cells per well and allowed to adhere. After adherence, the cells were rinsed twice with PBS to remove non‑adherent cells and then exposed to the indicated conditions for 48 h. Following this treatment, the cells were harvested, resuspended, and seeded into the inserts of a 24‑well plate containing culture‑inserts (ibidi80406); each insert received 100 μL of cell suspension at a density of 2 × 10^4^cells. After attachment, the inserts were carefully removed, and the wells were gently rinsed with PBS to eliminate non‑adherent cells. Images were acquired immediately (0 h) and again after 18 h of incubation. Migrated cells were observed and photographed using an inverted phase‑contrast microscope, with at least three random fields captured per condition. The migration gap was quantified by measuring intercellular distances using Image J software.

### Lactylome analysis

Global protein lactylation profiling was performed using affinity enrichment of lactylated peptides followed by liquid chromatography–tandem mass spectrometry. Proteins were digested with trypsin, and lactylated peptides were enriched using lactylation-specific affinity resin before separation on a NanoElute UHPLC system and analysis using a timsTOF Pro mass spectrometer in PASEF mode. Detailed sample preparation, affinity enrichment, chromatographic conditions, and mass spectrometric parameters are provided in the Supplementary Material 3.

### RNA sequencing and bioinformatics analysis

Primary Müller cells expressing MBD2-WT or MBD2-K186R were cultured under high-glucose conditions (25.5 mM), with six biologically independent samples in each group. RNA-seq libraries were sequenced on an Illumina NovaSeq 6000 platform to generate 150-bp paired-end reads. After quality filtering using fastp, clean reads were aligned to the reference genome using HISAT2, and gene-level read counts were obtained using featureCounts. Differential expression analysis was performed using DESeq2, with an adjusted *P* value < 0.05 and a fold change > 2 or < 0.5 used to define differentially expressed genes. Principal component analysis, volcano plot visualization, and gene set enrichment analysis were subsequently performed. Detailed library preparation and bioinformatics procedures are provided in the Supplementary Material 3.

### Statistical analyses

Continuous variables were assessed for normality using the Shapiro–Wilk test and are presented as mean ± standard deviation (SD) or median (interquartile range [IQR]), as appropriate. Homogeneity of variance was evaluated using Levene’s test, with Welch’s correction applied when necessary.

Two-group comparisons were performed using an unpaired two-tailed Student’s t-test for normally distributed data, with Welch’s correction when variances were unequal, or the Mann–Whitney U test for non-normally distributed data. Categorical variables were analyzed using the chi-square test or Fisher’s exact test, as appropriate. For comparisons among three or more groups, one-way ANOVA or the Kruskal–Wallis test was used according to data distribution, followed by pairwise comparisons. *P* values from multiple comparisons were adjusted using the Benjamini–Hochberg procedure to control the false discovery rate.

All tests were two-sided. A *P* value < 0.05, or a Benjamini–Hochberg-adjusted *P* value < 0.05 for multiple comparisons, was considered statistically significant. Statistical analyses and graph generation were performed using GraphPad Prism 9, with additional analyses conducted using IBM SPSS Statistics version 22.0 for Mac.

## Supplementary Information

Supplementary Material 1.

Supplementary Material 2.

Supplementary Material 3.

## Data Availability

The RNA sequencing data generated in this study have been deposited in the National Center for Biotechnology Information (NCBI)-Gene Expression Omnibus (GEO) database (GSE347442). The raw mass spectrometry data of the lysine lactylation proteomics analysis have been deposited in the ProteomeXchange Consortium via the iProX repository (PXD084476). Requests for other data access should be directed to the corresponding authors and will be subject to institutional review board approval and execution of appropriate data use agreements to ensure patient confidentiality and compliance with applicable privacy regulations.

## References

[1] GBD 2019 Blindness and Vision Impairment Collaborators, Vision Loss Expert Group of the Global Burden of Disease Study. Trends in prevalence of blindness and distance and near vision impairment over 30 years: an analysis for the Global Burden of Disease Study. Lancet Glob Health. 2021;9(2):e130–43. 10.1016/S2214-109X(20)30425-3.

[2] Yau JWY, Rogers SL, Kawasaki R, Lamoureux EL, Kowalski JW, Bek T, et al. Global prevalence and major risk factors of diabetic retinopathy. Diabetes Care. 2012;35(3):556–64. 10.2337/dc11-1909. PMC3322721 22301125

[3] Antonetti DA, Klein R, Gardner TW. Diabetic retinopathy. N Engl J Med. 2012;366(13):1227–39. 10.1056/NEJMra1005073. 22455417

[4] Wong TY, Cheung CMG, Larsen M, Sharma S, Simó R. Diabetic retinopathy. Nat Rev Dis Primers. 2016;2:16012. 10.1038/nrdp.2016.12. 27159554

[5] Walshe R, Esser P, Wiedemann P, Heimann K. Proliferative retinal diseases: myofibroblasts cause chronic vitreoretinal traction. Br J Ophthalmol. 1992;76(9):550–2. 10.1136/bjo.76.9.550. PMC504347 1420061

[6] Yang S, Qi S, Wang C. The role of retinal Müller cells in diabetic retinopathy and related therapeutic advances. Front Cell Dev Biol. 2022;10:1047487. 10.3389/fcell.2022.1047487. PMC9757137 36531955

[7] Coughlin BA, Feenstra DJ, Mohr S. Müller cells and diabetic retinopathy. Vision Res. 2017;139:93–100. 10.1016/j.visres.2017.03.013. PMC5794018 28866025

[8] Guidry C, King JL, Mason JO 3rd. Fibrocontractive Müller cell phenotypes in proliferative diabetic retinopathy. Invest Ophthalmol Vis Sci. 2009;50(4):1929–39. 10.1167/iovs.08-2475. 19117921

[9] Bringmann A, Wiedemann P. Müller glial cells in retinal disease. Ophthalmologica. 2012;227(1):1–19. 10.1159/000328979. 21921569

[10] Wu D, Kanda A, Liu Y, Noda K, Murata M, Ishida S. Involvement of Müller glial autoinduction of TGF-β in diabetic fibrovascular proliferation via glial–mesenchymal transition. Invest Ophthalmol Vis Sci. 2020;61(14):29. 10.1167/iovs.61.14.29. PMC7774059 33369638

[11] Fan J, Shen W, Lee SR, Mathai AE, Zhang R, Xu G, et al. Targeting the Notch and TGF-β signaling pathways to prevent retinal fibrosis in vitro and in vivo. Theranostics. 2020;10(18):7956–73. 10.7150/thno.45192. PMC7381727 32724452

[12] Yi C, Liu J, Deng W, Luo C, Qi J, Chen M, et al. Old age promotes retinal fibrosis in choroidal neovascularization through circulating fibrocytes and profibrotic macrophages. J Neuroinflammation. 2023;20(1):45. 10.1186/s12974-023-02731-y. PMC9947907 36823538

[13] Winkler BS, Arnold MJ, Brassell MA, Puro DG. Energy metabolism in human retinal Müller cells. Invest Ophthalmol Vis Sci. 2000;41(10):3183–90. PMC1766465 10967082

[14] Magistretti PJ, Allaman I. Lactate in the brain: from metabolic end-product to signalling molecule. Nat Rev Neurosci. 2018;19(4):235–49. 10.1038/nrn.2018.19. 29515192

[15] Brown TP, Ganapathy V. Lactate/GPR81 signaling and proton motive force in cancer: role in angiogenesis, immune escape, nutrition, and Warburg phenomenon. Pharmacol Ther. 2020;206:107451. 10.1016/j.pharmthera.2019.107451. 31836453

[16] Madaan A, Chaudhari P, Nadeau-Vallée M, Hamel D, Zhu T, Mitchell G, et al. Müller cell-localized G-protein-coupled receptor 81 (hydroxycarboxylic acid receptor 1) regulates inner retinal vasculature via Norrin/Wnt pathways. Am J Pathol. 2019;189(9):1878–96. 10.1016/j.ajpath.2019.05.016. 31220454

[17] Kottmann RM, Kulkarni AA, Smolnycki KA, Lyda E, Dahanayake T, Salibi R, et al. Lactic acid is elevated in idiopathic pulmonary fibrosis and induces myofibroblast differentiation via pH-dependent activation of transforming growth factor-β. Am J Respir Crit Care Med. 2012;186(8):740–51. 10.1164/rccm.201201-0084OC. PMC3480515 22923663

[18] Xie N, Tan Z, Banerjee S, Cui H, Ge J, Liu RM, et al. Glycolytic reprogramming in myofibroblast differentiation and lung fibrosis. Am J Respir Crit Care Med. 2015;192(12):1462–74. 10.1164/rccm.201504-0780OC. PMC4731722 26284610

[19] Zhang D, Tang Z, Huang H, Zhou G, Cui C, Weng Y, et al. Metabolic regulation of gene expression by histone lactylation. Nature. 2019;574(7779):575–80. 10.1038/s41586-019-1678-1. PMC6818755 31645732

[20] Cui H, Xie N, Banerjee S, Ge J, Jiang D, Dey T, et al. Lung myofibroblasts promote macrophage profibrotic activity through lactate-induced histone lactylation. Am J Respir Cell Mol Biol. 2021;64(1):115–25. 10.1165/rcmb.2020-0360OC. PMC7780997 33074715

[21] Rho H, Terry AR, Chronis C, Hay N. Hexokinase 2-mediated gene expression via histone lactylation is required for hepatic stellate cell activation and liver fibrosis. Cell Metab. 2023;35(8):1406-1423.e8. 10.1016/j.cmet.2023.06.013. PMC11748916 37463576

[22] Zong Z, Xie F, Wang S, Wu X, Zhang Z, Yang B, et al. Alanyl-tRNA synthetase, AARS1, is a lactate sensor and lactyltransferase that lactylates p53 and contributes to tumorigenesis. Cell. 2024;187(10):2375-2392.e33. 10.1016/j.cell.2024.04.002. 38653238

[23] Hendrich B, Bird A. Identification and characterization of a family of mammalian methyl-CpG binding proteins. Mol Cell Biol. 1998;18(11):6538–47. 10.1128/MCB.18.11.6538. PMC109239 9774669

[24] Ge Y, Zhang R, Feng Y, Lu J, Li H. Mbd2 deficiency alleviates retinal cell apoptosis via the miR-345-5p/Atf1 axis in high glucose injury and streptozotocin-induced diabetic mice. Mol Ther Nucleic Acids. 2021;26:1201–14. 10.1016/j.omtn.2021.10.026. PMC8605293 34853720

[25] Wang Y, Zhang L, Wu GR, Zhou Q, Yue H, Rao LZ, et al. MBD2 serves as a viable target against pulmonary fibrosis by inhibiting macrophage M2 program. Sci Adv. 2021;7(1):eabb6075. 10.1126/sciadv.abb6075. PMC7775789 33277324

[26] Wang Y, Zhang L, Huang T, Wu GR, Zhou Q, Wang FX, et al. The methyl-CpG-binding domain 2 facilitates pulmonary fibrosis by orchestrating fibroblast to myofibroblast differentiation. Eur Respir J. 2022;60(3):2003697. 10.1183/13993003.03697-2020. PMC9520034 35086828

[27] Yu FX, Guan KL. The Hippo pathway: regulators and regulations. Genes Dev. 2013;27(4):355–71. 10.1101/gad.210773.112. PMC3589553 23431053

[28] Hamon A, Masson C, Bitard J, Gieser L, Roger JE, Perron M. Retinal degeneration triggers the activation of YAP/TEAD in reactive Müller cells. Invest Ophthalmol Vis Sci. 2017;58(4):1941–53. 10.1167/iovs.16-21366. PMC6024660 28384715

[29] Zhang W, Kong Y. YAP is essential for TGF-β-induced retinal fibrosis in diabetic rats via promoting the fibrogenic activity of Müller cells. J Cell Mol Med. 2020;24(21):12390–400. 10.1111/jcmm.15739. PMC7686973 32951332

[30] Dupont S, Morsut L, Aragona M, Enzo E, Giulitti S, Cordenonsi M, et al. Role of YAP/TAZ in mechanotransduction. Nature. 2011;474(7350):179–83. 10.1038/nature10137. 21654799

[31] Panciera T, Azzolin L, Cordenonsi M, Piccolo S. Mechanobiology of YAP and TAZ in physiology and disease. Nat Rev Mol Cell Biol. 2017;18(12):758–70. 10.1038/nrm.2017.87. PMC6192510 28951564

[32] Min J, Zeng T, Roux M, Lazar D, Chen L, Tudzarova S. The role of HIF1α-PFKFB3 pathway in diabetic retinopathy. J Clin Endocrinol Metab. 2021;106(9):2505–19. 10.1210/clinem/dgab362. PMC8372643 34019671

[33] Tomita Y, Cagnone G, Fu Z, Cakir B, Kotoda Y, Asakage M, et al. Vitreous metabolomics profiling of proliferative diabetic retinopathy. Diabetologia. 2021;64(1):70–82. 10.1007/s00125-020-05309-y. PMC7718434 33099660

[34] Shen Y, Jiang L, Wen P, Ye Y, Zhang Y, Ding H, et al. Tubule-derived lactate is required for fibroblast activation in acute kidney injury. Am J Physiol Renal Physiol. 2020;318(3):F689–701. 10.1152/ajprenal.00229.2019. 31928224

[35] Leighton GO, Irvin EM, Kaur P, Liu M, You C, Bhattaram D, et al. Densely methylated DNA traps methyl-CpG-binding domain protein 2 but permits free diffusion by methyl-CpG-binding domain protein 3. J Biol Chem. 2022;298(10):102428. 10.1016/j.jbc.2022.102428. PMC9520026 36037972

[36] Stirzaker C, Song JZ, Ng W, Du Q, Armstrong NJ, Locke WJ, et al. Methyl-CpG-binding protein MBD2 plays a key role in maintenance and spread of DNA methylation at CpG islands and shores in cancer. Oncogene. 2017;36(10):1328–38. 10.1038/onc.2016.297. 27593931

[37] Ai K, Pan J, Zhang P, Li H, He Z, Zhang H, et al. Methyl-CpG-binding domain protein 2 contributes to renal fibrosis through promoting polarized M1 macrophages. Cell Death Dis. 2022;13(2):125. 10.1038/s41419-022-04577-3. PMC8826408 35136032

[38] Noguchi S, Saito A, Nagase T. YAP/TAZ signaling as a molecular link between fibrosis and cancer. Int J Mol Sci. 2018;19(11):3674. 10.3390/ijms19113674. PMC6274979 30463366

[39] Gui Y, Li J, Lu Q, Feng Y, Wang M, He W, et al. Yap/Taz mediates mTORC2-stimulated fibroblast activation and kidney fibrosis. J Biol Chem. 2018;293(42):16364–75. 10.1074/jbc.RA118.004073. PMC6200934 30154246

[40] Toyama T, Looney AP, Baker BM, Stawski L, Haines P, Simms R, et al. Therapeutic targeting of TAZ and YAP by dimethyl fumarate in systemic sclerosis fibrosis. J Invest Dermatol. 2018;138(1):78–88. 10.1016/j.jid.2017.08.024. PMC5742036 28870693

[41] Lei Y, Liu Q, Chen B, Wu F, Li Y, Dong X, et al. Protein O-GlcNAcylation coupled to Hippo signaling drives vascular dysfunction in diabetic retinopathy. Nat Commun. 2024;15(1):9334. 10.1038/s41467-024-53601-x. PMC11522279 39472558

