# Supplementary material for: Lactate-induced MBD2 K186 lactylation promotes YAP1-dependent myofibroblast-like remodeling of Müller cells in diabetic retinopathy
Source: Mol Biomed. 2026 Oct 6;7:197. doi: 10.1186/s43556-026-00605-2 (PMC13638938; doi:10.1186/s43556-026-00605-2)
Supplement: Supplementary file 1 [file 43556_2026_605_MOESM3_ESM.docx]

**Lactate-induced MBD2 K186 lactylation promotes YAP1-dependent myofibroblast-like remodeling of Müller cells in diabetic retinopathy**

Xindan Xing**^†^** ^1^, Yan Jiang**^†^**^1^, Junwei Fang**^†^** ^1^, Xin Shi^1^, Qian Zhu^1^, Yinchen shen^1^, Li Su^1^, Xinyi Liu^1^, Hanying Wang^1^, Jin Wei^1^, Yuan Qu^1^, Kangjia Lv^1^, Yu Xiao^1^, Jingtong Xie^1^, Xiaoxin Liu^1^, Hancong Wan^1^, Tian Niu* ^1^, Kun Liu* ^1^

1：Department of Ophtalmology, Shanghai General Hospital, National Clinical Research Center for Eye Diseases, Shanghai Clinical Research Center for Eye Diseases, Shanghai Key Clinical Specialty, Shanghai Key Laboratory of Ocular Fundus Diseases, Shanghai Engineering Center for Visual Science and Photomedicine,Shanghai engineering center for precise diagnosis and treatment of eye disease, Shanghai, China.

#:Xindan Xing, Yan Jiang and Junwei Fang contributed equally to this work.

*: Corresponding authors

Kun Liu: [drliukun@sjtu.edu.cn](mailto:drliukun@sjtu.edu.cn), 0000-0002-0115-7160

Tian Niu: niutian_up@163.com, 0000-0002-0377-3151

**Supplementary Methods**

1．**Human vitreous specimens processing**

Samples were thawed at 4°C for metabolite extraction. A 50 μL sample was combined with 200 μL of pre-chilled chloroform methanol (2:1, v/v), vortexed for 30 sec, incubated at room temperature for 5 min, and centrifuged at 13,000×g for 15 min. Upper and lower phases were collected separately and vacuum‑dried at room temperature.

2．**Liquid chromatography‑mass spectrometry (LC‑MS) metabolomics**

Metabolites were separated using an Ultimate 3000 UHPLC system (Thermo Fisher Scientific, USA) with column of HSS T3 (2.1 mm i.d. × 100 mm, 1.8 μm; USA) at 35°C. Mobile phase A was 0.1% formic acid (v/v); mobile phase B was acetonitrile. Flow rate: linear gradient 0.3 mL/min: 0 min, 2% B; 1.5 min, 2% B; 12 min, 100% B; 15 min, 100% B. Injection volume: 1 μL. Ion detection used Q-Exactive MS (Thermo Scientific, USA) with electrospray source in ion switching mode. Data were collected in full scan mode. Acquisition parameters include +3.5/−4.0 kV, 300°C, 40 and 10 arbitrary units sheath and auxiliary gas, respectively. The m/z range is set from 200 - 2000.

3．**Real-time quantitative PCR (qRT-PCR)**

Total RNA was extracted using Trizol per manufacturer’s protocol. RNA was reverse-transcribed into cDNA using PrimeScript RT Master Mix (Takara Bio, Japan) for mRNA analysis. mRNA expression was quantified using qPCR with SYBR Premix Ex Taq (Takara Bio, Japan) on an ABI 7500HT (Applied Biosystems, USA), employing β-actin as the internal control. Primer sequences are in supplementary information (Supplementary Table S1).

4．**Western blotting**

Retinal samples and cell samples were lysed in Western and IP Lysis buffer (Beyotime, China), with protein concentration quantified via BCA assay (Beyotime, China). Equal amounts of extracted proteins were resolved on 4% ‑ 20% SDS-PAGE (Genscript Biotech Corporat, Chnia) then transferred to Nitrocellulose (NC) membranes (Thermo Fisher Scientific, USA) at 110 V for 60 min. Then blocked the bolts using 5% milk in TBST for 30 min, incubated with specific primary antibodies over 8 h at 4°C. Band densitometry was analyzed using Image-J (public domain, NIH, USA). Triplicate raw values were averaged; all bands were normalized to respective β-actin or β-tubulin controls and untreated controls. Antibody details are listed in supplementary information (Supplementary Table S2).

5．**Immunostaining**

For cryosections: eyeballs were enucleated and fixed via 4% paraformaldehyde (PFA) (v/v) at 4°C for 1‑2 h, washed lightly in PBS. After cryoprotection with 30% sucrose in PBS over 8 h at 4°C refrigerator, retinas were inserted in optimal cutting temperature (OCT) and flash-frozen in liquid nitrogen. Sections (10‑12 μm) were cut using cryostat (Leica Biosystems). For whole mounts: post-enucleation, eyeballs were incised at the limbus; retinas were carefully dissected, freed from vitreous, and flat-mounted (ganglion cell layer up) on nitrocellulose filters. They were fixed in 4% PFA (v/v), 30 min, 4°C , washed lightly in PBS. Briefly, blocked the sections with 5% donkey serum (v/v) for 1 h at room temperature, then incubated over 8 h at 4°C using primary antibodies. Then incubated the sections with secondary antibodies at room temperature for 1 h. DAPI was used to stained nuclei. Antibody information are provided in supplementary information (Supplementary Table S2).

6．**Lactylome analysis**

Samples from -80°C were sonicated for lysis; protein concentration was quantified via BCA. Equal proteins were adjusted to consistent volume with lysis buffer, mixed with 20% final TCA, vortexed, and precipitated at 4°C for 2 h. After centrifugation (4500 g, 5 min), supernatant was discarded; precipitates were washed 2-3× with pre-chilled acetone. Dried precipitates were resuspended in 200 mM TEAB (sonicated to disrupt), then digested overnight with trypsin (1:50 protease:protein, m/m). Samples were reduced with 5 mM DTT (56°C, 30 min), then alkylated with 11 mM IAM (15 min, dark, RT). Peptides in IP buffer (100 mM NaCl, 1 mM EDTA, 50 mM Tris-HCl, 0.5% NP-40, pH 8.0) were incubated overnight with pre-washed lactylation resin (PTM-1404, Jingjie PTM Biolabs) on a 4°C rotator. Resin was washed 4× with IP buffer, 2× with water; bound peptides were eluted 3× with 0.1% trifluoroacetic acid, pooled, and vacuum-dried. Desalted via C18 ZipTips (per protocol), dried samples were reconstituted in LC mobile phase A (0.1% formic acid, 2% acetonitrile) and separated on a NanoElute UHPLC. Gradient: 0−40 min (6%−24% B), 40−54 min (24%−35% B), 54−57 min (35%−80% B), 57−60 min (80% B); flow 450 nl/min (B:0.1% formic acid in acetonitrile). Peptides were ionized (2.0 kV) and analyzed on a timsTOF Pro MS. PASEF mode: 1 full MS scan + 10 MS/MS scans (precursors, charge 0-5); dynamic exclusion 30 s. MS/MS range: 100-1700 m/z.

7．**RNA sequencing and bioinformatics analysis**

Primary Müller cells expressing MBD2-WT or MBD2-K186R were cultured under high-glucose conditions (25.5 mM), with six biologically independent samples included in each group. Total RNA was extracted using the TRIzol reagent (Invitrogen, CA, USA) according to the manufacturer’s protocol. Then the libraries were constructed using VAHTS Universal V6 RNA-seq Library Prep Kit according to the manufacturer’s instructions. The transcriptome sequencing and analysis were conducted by OBiO Technology Corp., Ltd. (Shanghai, China). The libraries were sequenced on an llumina Novaseq 6000 platform and 150 bp paired-end reads were generated. Raw reads were firstly processed using fastp (v0.22.0) and the low quality reads were removed to obtain the clean reads. The clean reads were mapped to the reference genome using HISAT2 (v2.1.0). FPKM of each gene was calculated and the read counts of each gene were obtained by featureCounts. PCA analysis were performed using R (v 3.2.0) to evaluate the biological duplication of samples. Differential expression analysis was performed using the DESeq2. The padj < 0.05 and Fold change > 2 or fold change < 0.5 was set as the threshold for significantly differential expression gene (DEGs). The analysis was used a predefined gene set, and the genes were ranked according to the degree of differential expression in the two types of samples. Then it is tested whether the predefined gene set was enriched at the top or bottom of the ranking list. Differential expression was visualized using volcano plots.

**Supplementary Figures**

**Figure S1**

**Figure S1. GPR81 and MCT1 are expressed in retinal Müller cells**
(a, b) Representative immunofluorescence images of retinal sections showing co-localization of glutamine synthetase (GS, green), a Müller cell marker, with GPR81 (a, red) or monocarboxylate transporter 1 (MCT1; b, red). Nuclei were counterstained with DAPI (blue). Merged images show the spatial distribution of GPR81 and MCT1 relative to GS-positive Müller cells. Boxed regions are shown at higher magnification in the corresponding zoomed images. Images are representative of three independent experiments (n = 3). Scale bars: 50 μm in the original images and 20 μm in the magnified images of panels a and b.

**Figure S2**

**Figure S2. Characterization and purity assessment of primary Müller cell cultures**
(a–c) Representative immunofluorescence images used to characterize primary Müller cell cultures. (a) Cells were stained for the Müller cell marker GLAST (red), together with the retinal ganglion cell marker RBPMS (green) and the endothelial cell marker CD31 (magenta). (b) Cells were stained for the Müller cell marker CRALBP (red) and vimentin (green), together with the microglial marker IBA1 (magenta). (c) Cells were co-stained for the Müller cell markers aquaporin-4 (AQP4, green) and glutamine synthetase (GS, red). Nuclei were counterstained with DAPI (blue). Scale bars: 100 μm. (d, e) Representative flow-cytometric analysis of primary Müller cell cultures showing the proportions of CRALBP-positive (d, 95.8%) and GS-positive (e, 95.4%) cells, supporting the high purity of the isolated Müller cell population. Images and flow-cytometric plots are representative of three independent experiments (n = 3).

**Figure S3**

**Supplementary Fig. S3 Verification of MBD2-WT, MBD2-K186R, and YAP1 overexpression in Müller cells.**
a, Representative western blots confirming the expression of FLAG-tagged wild-type MBD2 (MBD2-WT) and the K186R mutant (MBD2-K186R) in primary Müller cells following viral transduction. FLAG and MBD2 were detected with the indicated antibodies, and β-actin served as the loading control (n = 3 biologically independent experiments).
b, Representative western blot confirming YAP1 overexpression in primary Müller cells transduced with the YAP1-overexpression vector (OE-YAP1) compared with the negative-control vector (OE-NC); β-actin served as the loading control (n = 3 biologically independent experiments).

**Figure S4 Schematic maps of plasmid and viral constructs used in this study**

**Figure S4. Schematic maps of plasmid and viral constructs used in this study.** (a) Lentiviral vector expressing wild-type MBD2 (MBD2-WT). (b) Lentiviral vector expressing the MBD2 K186R mutant. (c) YAP1 overexpression plasmid. (d) Empty vector control plasmid**.**

**Supplementary Tables**

**Table S1 Characteristics of Study Population for Vitreous Metabolomics**

| Characteristic | PDR ( n = 106 ) | Non-DR ( n= 72 ) | P value |
| --- | --- | --- | --- |
| Age^‡^ (y) | 64 ± 6 | 54 ± 16 | <0.001 |
| Gender^†^ (male/female), n | 56/50 | 29/43 | 0.100 |
| Fasting Blood Glucose^‡^ (mmol/L) | 4.8 ± 0.55 | 6.6 ± 1.7 | <0.001 |
| Hypertension^†^ (yes/no) | 60/46 | 34/38 | 0.219 |
| Total cholesterol^‡^ (mmol/L) | 4.69 ± 0.43 | 4.58 ± 1.80 | 0.286 |
| Triglyceride^‡^ (mmol/L) | 1.54 ± 0.86 | 1.34 ± 1.02 | 0.068 |
| Treatments, n |  |  |  |
| Anti-VEGF^†^ (yes/no) | 34/72 | 0/72 | <0.001 |
| Antidiabetic medications^†^ (yes/no) | 106/0 | 0/72 | <0.001 |
| Antihypertension† (yes/no) | 58/48 | 30/42 | 0.087 |
| Age, total cholesterol, fasting blood glucose and triglyceride are represented as median ± interquartile range.  †Chi-square test.  ‡Mann–Whitney U test. | | | |

**Table S2. Primer sequences used for qPCR.**

| **Sequence 5’-3’** | |
| --- | --- |
| ACTA2-F: GCCGCCGATCCAGACAGAA | ACTA2-R: ATGACCCAGATTATGTTTGAGACC |
| TAGLN-F: TGTGAACTCCCTCTTATGCT | TAGLN--R: GAGGACTGTAATGGCTTTGG |
| COL1A1-F:AGAAGTCATAGGAGTCGAGGGA | COL1A1-R:CATAGGACATCTGGGAAGCAA |
| β-actin-F:ACGTTGACATCCGTAAAGAC | β-actin-R:GAAGGTGGACAGTGAGGC |

**Table S3. Primary antibodies used in this study.**

| **Antibody** | **catalog number & company** |
| --- | --- |
| GS | ab64713, Abcam |
| GFAP | ab4674, Abcam |
| Vimentin | ab92547, Abcam |
| CD31 | ab222783, Abcam |
| IBA-1 | ab178846, Abcam |
| RBPMS | ab152101, Abcam |
| α-SMA | ab124964, Abcam |
| α-SMA | AC220829016, Servicebio |
| SM22 | ab14106, Abcam |
| COL1A1 | R26615, Zenbio |
| YAP1 | 66900-10-Ig, proteintech |
| Pan-kla | PTM-1401RM, PTM Biolabs Inc |
| MBD2 | ab188474, Abcam |
| FLAG | SAB4200071, Sigma-Aldrich |
| β-Actin | H17371, Yeasen biotechnology |
| β-Tubulin | H6927690, Yeasen biotechnology |
| HRP-labeled Goat Anti-Mouse IgG | A0216, Beyotime |
| HRP-labeled Goat Anti-Rabbit IgG | A0208, Beyotime |
| AffiniPure® Goat Anti-Mouse IgG, light chain specific | [115-005-174](https://www.jacksonimmuno.com/catalog/products/115-005-174), Jackson |
| IgG Fraction Monoclonal Mouse Anti-Rabbit  IgG, light chain specific | [211-002-171](https://www.jacksonimmuno.com/catalog/products/211-002-171), Jackson |
| Donkey anti-rabbit Alexa Fluor 594 antibody | 711-585-152, Jackson ImmunoResearch |
| Donkey anti-mouse Alexa Fluor 488 antibody | 716-540-020, Jackson ImmunoResearch |
| Goat anti-chicken Alexa Fluor 488 antibody | 305-545-006, Jackson ImmunoResearch |
| CRALBP | 15356-1-AP, Proteintech |
| CRALBP | HA721330, HUABOI |
| GS | 113027-2-AP, Proteintech |
| AQP4 | 16473-1-AP, Proteintech |
| P-YAP1(Ser127） | 80694-2-RR, Proteintech |
| Secondary antibody (FITC-labeled goat anti-rabbit) | GB22303, Servicebio |
| Abcam Goat Anti-Rabbit IgG H&L (APC) preadsorbed | ab130805, Abcam |
